# Sex Disparities in Cardiogenic Shock: Risk Factors, Treatment Intensity, and Mortality in a Single Latin American Country

**DOI:** 10.5334/gh.1469

**Published:** 2025-09-09

**Authors:** Alexandra Arias-Mendoza, Héctor González-Pacheco, Amada Álvarez-Sangabriel, Diego Araiza-Garaygordobil, Pamela Ramírez-Rangel, Rodrigo Gopar-Nieto, Maria del Carmen López-Rodríguez, Daniel Sierra-Lara-Martínez, Salvador Mendoza-García, Braiana Ángeles Díaz-Herrera, María Nila Papaqui-Quitl, Jaime Hernández-Montfort, Jorge A. Ortega-Hernández

**Affiliations:** 1 Instituto Nacional de Cardiología Ignacio Chávez, Coronary Care Unit, Juan Badiano 1, Sección XVI, Tlalpan 14080 Ciudad De México, México; 2 Instituto Nacional de Cardiología Ignacio Chávez, Heart Failure Clinic, Juan Badiano 1, Sección XVI, Tlalpan 14080 Ciudad De México, México; 3 Instituto Nacional de Cardiología Ignacio Chávez, Cardiology Department, Juan Badiano 1, Sección XVI, Tlalpan 14080 Ciudad De México, México; 4 Advanced Heart Failure and Recovery Program for Central Texas Baylor Scott & White Health, 302 University Blvd Round Rock, TX 78665, USA

**Keywords:** Cardiogenic shock, sex disparities, acute myocardial infarction, treatment intensity

## Abstract

**Background:**

Cardiogenic shock (CS) carries a high in-hospital mortality, with limited data on sex-related disparities in Latin America. Women remain underrepresented in CS studies.

**Objectives:**

To evaluate sex-specific differences in characteristics, management, and mortality in acute myocardial infarction–related (AMI-CS) and non-AMI-CS in a large Latin-American cohort.

**Methods:**

We retrospectively analyzed 9430 patients (5016 AMI-CS and 4414 non-AMI-CS) with SCAI-CSWG stages B–E in a reference center in Mexico City from 2005 to 2023. The primary outcome was in-hospital mortality. Analyses included multivariable Cox models and propensity score matching (PSM).

**Results:**

Women with AMI-CS were older (67 vs. 60 years), had more hypertension (66% vs. 52%) and diabetes (53% vs. 38%), and received less primary reperfusion (62% vs. 71%) and mechanical circulatory support (11.6% vs. 14.7%) than men (all *P* < 0.05). In non-AMI-CS, women were older (66 vs. 60 years), had more prior heart failure (33% vs. 24%), while men had more chronic obstructive pulmonary disease (COPD) and prior MI (all *P* < 0.05). Unadjusted mortality was higher in women in AMI-CS (24.6% vs. 16.3%, HR 1.48, 95% CI 1.28–1.72) and non-AMI-CS (HR 1.18, 95% CI 1.05–1.32). After PSM, mortality differences were not significant in AMI-CS (HR 1.22, 95% CI 1.00–1.48) or non-AMI-CS (HR 1.07, 95% CI 0.92–1.24).

**Conclusions:**

Women with CS in Latin America present with greater comorbidity and less aggressive/invasive management. While unadjusted mortality was higher in women, these differences were no longer significant after PSM, indicating that baseline factors and treatment disparities largely explain excess risk.

**Central Illustration:**
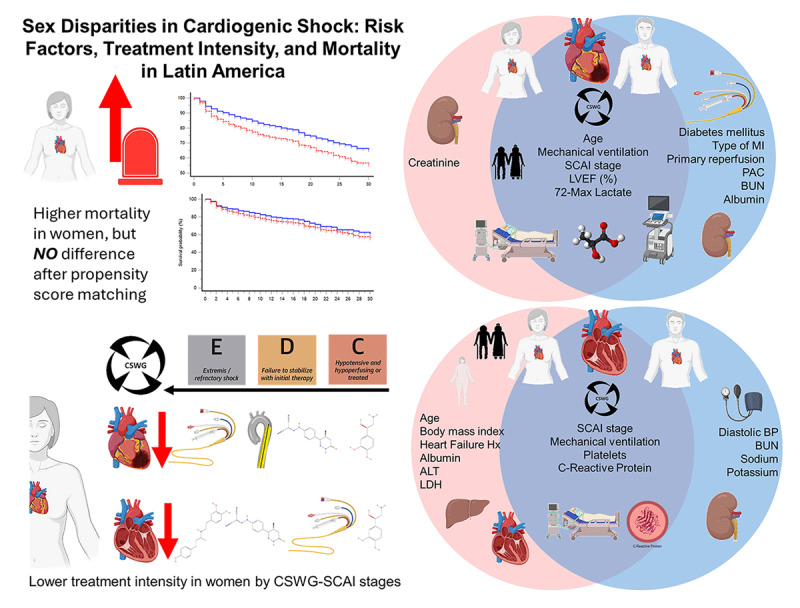
Sex disparities in cardiogenic shock: Risk factors, treatment intensity, and mortality in Latin America. Women presented higher mortality but had less treatment intensity among SCAI C or higher stages. Also, in the circles, we looked at similar (cross-over areas) and different (blue = men and red = women) risk factors for mortality.

## Background

Cardiogenic shock (CS) is defined as a sustained episode of at least one of the following: systolic blood pressure (SBP) <90 mm Hg for at least 30 min, use of vasoactive agents to maintain SBP, cardiac index <2.2 L/min/m^2^ in the absence of hypovolemia, or use of a mechanical support device (MCS) for clinically suspected CS. It occurs in ~5%–10% of acute myocardial infarction cases or advanced heart failure (HF) and carries hospital mortality rates of 30%–50% despite advances in care (1,2,3).

Data from Latin America are scarce, where disparities in access to reperfusion, mechanical circulatory support, and hemodynamic monitoring may contribute to different outcomes compared with high-income countries (4,5,6,7,8,9). The two main etiologies of CS are AMI-related CS and non-AMI-CS, which have a similar mortality rate (10,11).

Women tend to present at an elderly age and late to the hospital, thus having more MACEs and bleeding complications; also, they receive less primary reperfusion in AMI-CS. Furthermore, women with heart failure complicated by CS had higher vascular complications and bleeding rates (7). Women remain underrepresented in CS trials and often receive less MCS, with prior studies reporting inconsistent findings on sex differences in mortality – some showing higher mortality in women (6,7,8), others no difference (7,12,13).

Accordingly, we examined sex-based differences in clinical profile, treatment intensity, and in-hospital mortality in patients with AMI-CS and non-AMI-CS in a large Latin-American cohort. Beyond describing crude associations, our first objective was to identify subgroup-specific predictors of mortality. The second objective was to determine whether sex remained an independent determinant of risk after adjusting for demographic and clinical covariates, treatment strategies, and baseline imbalances.

## Methods

### Study design and setting

We analyzed a coronary care unit database (*n* = 28,054) from late 2015 to September 2023 at the Instituto Nacional de Cardiología Ignacio Chavez, which is an academic tertiary and reference center in Mexico City with a focus on only cardiovascular diseases from which we include patients with the diagnosis of CS was defined as Society for Cardiac Angiography and Interventions-Cardiogenic Shock Working Group (SCAI-CSWG) score (1), a sustained episode of at least 1 of the following: systolic blood pressure (SBP) <90 mmHg for at least 30 min, use of vasoactive agents to maintain SBP, cardiac index <2.2 L/min/m^2^ in the absence of hypovolemia, each determined to be secondary to cardiac dysfunction, or use of an MCS device for clinically suspected CS, and include those in SCAI B or higher maximum score at the first 72-h. No additional exclusion criteria were applied, except for patients without a confirmed diagnosis of cardiogenic shock.

The Research and Ethics Committee approved the study protocol; patient consent was waived because the study was retrospective and non-interventional. All procedures were conducted according to the Declaration of Helsinki and local regulations.

### Outcomes

The primary outcome was in-hospital mortality. Secondary analyses focused on: (1) identifying independent predictors of mortality within each subgroup (AMI-CS and non-AMI-CS, stratified by sex); and (2) evaluating whether sex was independently associated with mortality even after adjustment for demographics, comorbidities, treatment intensity, and other clinically relevant covariates.

## Statistical Analysis

Continuous variables were expressed as medians (interquartile range) and categorical variables as counts (percentages). Group comparisons were performed using the χ^2^ test for categorical variables and the Mann–Whitney U test for continuous variables.

Survival was assessed using Kaplan–Meier curves and 30-day restricted mean survival time (RMST), which was selected because early mortality events were frequent and the proportional hazards assumption may not always hold; thus, RMST provides an intuitive measure of average survival time within a fixed period. Our analytic strategy followed two sequential steps:

1. Identification of independent predictors

Cox proportional hazards regression was used to estimate hazard ratios (HRs) for in-hospital mortality. Variables with *P* < 0.05 in univariate analyses were entered into multivariable Cox models constructed separately for each sex and for AMI-CS and non-AMI-CS (Table S5). This step was intended to identify sex-specific independent predictors of mortality and to characterize the variables driving outcomes.

2. Adjustment for sex comparisons

To compare outcomes between sexes while minimizing confounding, adjusted Cox models and propensity score matching (PSM) were subsequently performed. Matching was conducted using the *K*-nearest neighbors method (*K* = 1, greedy algorithm) with a 1:1 ratio and a caliper width of 0.2. Matching variables included clinically relevant risk factors and treatment intensity measures, grouped as follows: age, body mass index (BMI), smoking history, hypertension, chronic obstructive pulmonary disease (COPD), prior heart failure, diabetes mellitus, chronic kidney disease, previous myocardial infarction, prior percutaneous coronary intervention, prior coronary artery bypass grafting, atrial fibrillation, pulmonary artery catheter (PAC) use, mechanical ventilation, hemodialysis, mechanical circulatory support (MCS), and SCAI-CSWG stage. For AMI-CS, myocardial infarction type and reperfusion strategy were also included. The final matched cohorts are presented in Supplementary Figure S3 and Table S5.

Treatment intensity levels were assigned and studied for an association with in-hospital mortality by sex in each CS etiology. All analyses were performed using IBM SPSS Statistics version 26 (IBM Corp., Armonk, NY) and SAS for Academics (SAS Institute Inc., Cary, NC). A two-sided *P*-value < 0.05 was considered statistically significant. Detailed methods are provided in the Supplementary Material.

## Results

**Figure 1:**
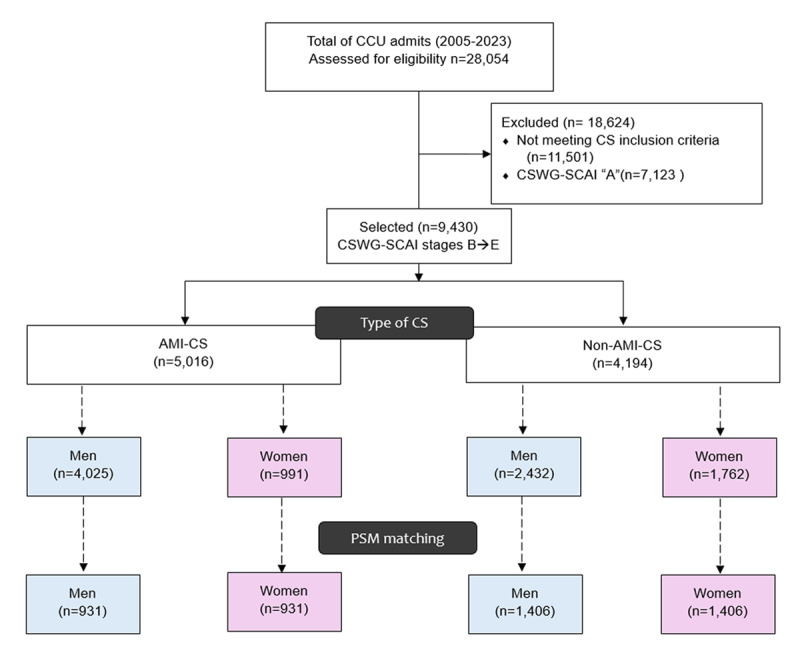
Patient flow diagram. Flow of patient screening, exclusions, and final cohorts with propensity score matching. AMI-CS, acute myocardial infarction–related cardiogenic shock; CS, cardiogenic shock; PSM, propensity score matching; CSWG-SCAI, cardiogenic shock working group-society for cardiovascular angiography and interventions.

We analyzed 9430 patients, SCAI-CSWG stage B or higher, maximum stage within the first 72 h: 4414 non-AMI-CS (2432 men vs. 1982 women) and 5016 AMI-CS (4025 men and 991 women; Figure 1).

### AMI cardiogenic shock

Women presented at an older age (67 vs. 60 years, *P* < 0.001), while men had higher BMI (*P* < 0.001). Hypertension and diabetes were more common in women (*P* < 0.001), whereas COPD and prior MI predominated in men (*P* = 0.005 for both). Women more often presented with NSTEMI (*P* < 0.001).

Cardiac and laboratory findings also differed. Women had slightly higher LVEF, but lower hemoglobin, estimated glomerular filtration (eGFR) by CKD-EPI, sodium, and chloride, with higher glucose (*P for all* <0.001). Liver enzymes were lower in women (AST/ALT, *P* < 0.001), while LDH was higher in men (*P* = 0.002). CRP did not differ. Minimum pH was lower in women (*P* < 0.001), though peak lactate was similar.

Management disparities were evident: reperfusion was less frequent in women (62% vs. 71%, *P* < 0.001), who also required more hemodialysis (*P* = 0.009) and multiple vasopressors, particularly norepinephrine (*P* < 0.001) and vasopressin (*P* = 0.006).

Additionally, MCS was more utilized in men (*P =* 0.011); specific use of intra-aortic balloon pump (IABP) was 14.7% in men vs. 11.6% in women (*P =* 0.012), but as of the date of the study, the low numbers of ECMO (8 men vs. 0 women) and Impella (2 men vs. 1 woman) were for the initiation of the utilization of these MCS in our media (*P* > 0.05).

Outcomes were worse in women, with higher AKI (*P* < 0.001), stroke (*P* = 0.03), and mortality (24.6% vs. 16.3%, P < 0.001). They were also more frequently classified in advanced SCAI-CSWG stages (*P* < 0.001; Table 1).

**Table 1:** Demographics, clinical parameters, management, and outcomes of patients with AMI and non-AMI cardiogenic shock, stratified by sex.


		AMI-CS			NON-AMI-CS		
	
		MEN (*n* = 4025)	WOMEN (*n* = 991)	P-VALUE	MEN (*n* = 2432)	WOMEN (*n* = 1982)	*P*-VALUE

Age (years)		60 (52–68)	67 (59–75)	<0.001	60 (46–71)	63 (50–75)	<0.001

Body mass index (kg/m^2^)		27.1 (24.8–29.69)	26.67 (23.88–29.64)	<0.001	25.71 (23.44–28.36)	25.33 (22.36–29.14)	0.012

Smoking history (%)		2708 (67.3)	320 (33.3)	<0.001	1289 (53)	320 (16.1)	<0.001

Dyslipidemia (%)		1201 (29.8)	298 (30.1)	0.886	490 (20.1)	253 (12.8)	<0.001

Hypertension (%)		1975 (49.1)	701 (70.7)	<0.001	1039 (42.7)	964 (48.6)	<0.001

COPD (%)		76 (1.9)	33 (3.3)	0.005	135 (5.6)	201 (10.1)	<0.001

Heart failure history (%)		349 (8.7)	107 (10.8)	0.037	1295 (53.2)	826 (41.7)	<0.001

Chronic kidney disease (%)		239 (5.9)	91 (9.2)	<0.001	408 (16.8)	251 (12.7)	<0.001

Diabetes mellitus (%)		1652 (41)	581 (58.6)	<0.001	600 (24.7)	511 (25.8)	0.398

Previous MI (%)		910 (22.6)	183 (18.5)	0.005	499 (20.5)	145 (7.3)	<0.001

Previous PCI (%)		498 (12.4)	79 (8)	<0.001	245 (10.1)	62 (3.1)	<0.001

Previous CABG (%)		102 (2.5)	34 (3.4)	<0.001	116 (4.8)	35 (1.8)	<0.001

Stroke history (%)		102 (2.5)	25 (2.5)	0.984	133 (5.5)	173 (8.7)	<0.001

Previous atrial fibrillation (%)		76 (1.9)	38 (3.8)	<0.001	445 (18.3)	650 (32.8)	<0.001

Type of ACS (%)	NSTEMI	1086 (27)	381 (38.4)	<0.001	(–)	(–)	NA
	
	STEMI	2939 (73)	610 (61.6)		(–)	(–)	

Admission vital signs and paraclinical work-up							

Systolic blood pressure (mmHg)		123 (110–140)	121 (100–146)	0.082	110 (90–130)	110 (90–130)	0.498

Diastolic blood pressure (mmHg)		77 (68–90)	72 (60–85)	<0.001	66 (60–80)	65 (59–80)	0.249

Medium arterial pressure (mmHg)		92.67 (81–106.67)	90 (74–104.33)	<0.001	80.67 (70–93.33)	80 (70–94.67)	0.917

Heart rate (bpm)		80 (70–98)	80 (68–98)	0.283	90 (70–110)	90 (65–110)	0.785

Respiratory rate (rpm)		18 (16–20)	18 (18–22)	<0.001	20 (18–24)	20 (18–24)	0.778

LVEF (%)		45 (35–54)	46 (35–55)	<0.001	40 (25–57)	54 (40–60)	<0.001

Hemoglobin (g/dL)		15.4 (14–16.6)	13 (11.5–14.3)	<0.001	14 (12–15.7)	12.6 (10.6–14.3)	<0.001

Leukocytes (c*10^9^ L)		11.6 (9.07–14.6)	11 (8.7–13.9)	<0.001	9.8 (7.5–13)	9.5 (6.9–13.4)	0.016

Neutrophils (%)		78.4 (70–85)	77.7 (68.15–84)	0.045	78 (69.7–85)	79 (69.4–86)	0.112

Platelets (c*10^9^ L)		222 (183–267)	252.5 (203–302)	<0.001	188 (143–244)	197 (142–263)	0.001

Glucose (mg/dL)		160 (124.9–234)	180 (131–269)	<0.001	120 (99–157)	126 (101–169)	<0.001

BUN (mg/dL)		19 (14.4–28)	22 (16–34)	<0.001	31 (20–53)	29 (18–49.39)	<0.001

Creatinine (mg/dL)		1.1 (0.9–1.45)	1 (0.79–1.5)	<0.001	1.46 (1.09–2.25)	1.2 (0.86–1.98)	<0.001

eGFR (mL/min/1.73 m^2^)		73.09 (49.88–91.79)	57.46 (34.73–82.44)	<0.001	51.75 (29.54–78.8)	48.21 (25.26–75.78)	0.001

Chloride (mEq/L)		103 (100–105.6)	102 (98.01–105)	<0.001	101 (96.7–105)	101 (96–105)	0.324

Sodium (mEq/L)		137 (134–139)	136 (133–139)	<0.001	135 (131–138.3)	135 (131–138.1)	0.164

Potassium (mEq/L)		4.2 (3.9–4.64)	4.3 (3.9–4.7)	0.164	4.44 (4–5.1)	4.4 (3.92–5.1)	0.05

Albumin (g/dL)		3.72 (3.4–4.03)	3.56 (3.19–3.87)	<0.001	3.4 (2.97–3.8)	3.4 (3–3.79)	0.301

AST (UI/L)		85 (36–243)	64 (30–192.61)	<0.001	37 (23–84.5)	34.9 (23–60.5)	0.002

ALT (UI/L)		51.4 (31–94.6)	41 (22.9–84.06)	<0.001	32 (19–74.4)	27 (17–52.6)	<0.001

Lactate dehydrogenase (UI/L)		484.95 (260–942)	432 (250–812)	0.002	340 (210.5–574.5)	340.5 (217.3–584)	0.279

C-reactive protein (mg/L)		13.5 (4.2–59.8)	14.6 (4.9–61.7)	0.216	28.7 (9.1–86.85)	22.74 (7.3–71.1)	<0.001

72h – minimum pH		7.4 (7.35–7.44)	7.38 (7.33–7.43)	<0.001	7.39 (7.32–7.44)	7.38 (7.31–7.43)	0.003

72h – maximum lactate		2.4 (2–3.3)	2.3 (2–3.5)	0.122	2.4 (2–3.6)	2.4 (1.9–3.6)	0.43

Cardiogenic shock management							

Primary reperfusion (%)	NR*	2553 (63.4)	717 (72.4)	<0.001	(–)	(–)	NA
	
	pPCI	1077 (26.8)	205 (20.7)		(–)	(–)	
	
	PI	395 (9.80)	69 (7.00)		(–)	(–)	

PAC (%)		283 (7.00)	66 (6.70)	0.681	64 (2.6)	25 (1.3)	0.001

Mechanical ventilation (%)		667 (16.6)	186 (18.8)	0.099	482 (19.8)	417 (21)	0.317

Hemodialysis (%)		140 (3.50)	52 (5.20)	0.009	157 (6.50)	119 (6)	0.538

Number of vasoactives	0	2744 (68.2)	603 (60.8)	<0.001	1390 (57.2)	1158 (58.4)	0.136
	
	1	489 (12.1)	157 (15.8)		498 (20.5)	417 (21)	
	
	2	362 (9.00)	113 (11.4)		349 (14.4)	283 (14.3)	
	
	3	333 (8.30)	88 (8.90)		162 (6.7)	110 (5.5)	
	
	4	97 (2.40)	30 (3.00)		33 (1.40)	14 (0.7)	

Levosimendan (%)		224 (5.60)	47 (4.70)	0.305	149 (6.1)	51 (2.6)	<0.001

Dobutamine (%)		819 (20.3)	228 (23.0)	0.065	548 (22.5)	312 (15.7)	<0.001

Norepinephrine (%)		1060 (26.3)	337 (34.0)	<0.001	782 (32.2)	694 (35.0)	0.045

Vasopressin (%)		497 (12.3)	155 (15.6)	0.006	335 (13.8)	312 (15.7)	0.066

MCS (%)		594 (14.8)	115 (11.6)	0.011	50 (2.1)	27 (1.4)	0.08

Cardiogenic shock outcomes							

SCAI (%)	E	567 (14.1)	174 (17.6)	<0.001	433 (17.8)	371 (18.7)	0.296
	
	D	392 (9.70)	112 (11.3)		312 (12.8)	218 (11.0)	
	
	C	573 (14.2)	172 (17.4)		522 (21.5)	428 (21.6)	
	
	B	2493 (61.9)	533 (53.8)		1165 (47.9)	965 (48.7)	

Mortality (%)		656 (16.3)	244 (24.6)	<0.001	586 (24.1)	536 (27)	0.025

Stroke (%)		46 (1.1)	20 (2)	0.03	31 (1.3)	29 (1.5)	0.591

VT/VF (%)		538 (13.4)	128 (12.9)	0.708	237 (9.7)	180 (9.1)	0.454

GIB (%)		93 (2.3)	30 (3)	0.191	49 (2)	45 (2.3)	0.558

PE (%)		0	1 (0.1)	0.198	8 (0.3)	7 (0.4)	0.891

AKI (%)		881 (21.9)	307 (31)	<0.001	866 (35.6)	784 (39.6)	0.007

Nosocomial pneumonia (%)		179 (4.4)	42 (4.2)	0.774	82 (3.4)	74 (3.7)	0.517

Sepsis (%)		160 (4)	41 (4.1)	0.816	192 (7.9)	154 (7.8)	0.878


*NR patients comprise NSTEMI patients or STEMI late presenters that did not have a primary reperfusion. ACS, acute coronary syndrome; AMI, acute myocardial infarction; AKI, acute kidney injury; ALT, alanine aminotransferase; AST, aspartate aminotransferase; BUN, blood urea nitrogen; CABG, coronary artery bypass grafting; COPD, chronic obstructive pulmonary disease; CRP, C-reactive protein; CS, cardiogenic shock; eGFR, estimated glomerular filtration rate; GIB, gastrointestinal bleeding; LVEF, left ventricular ejection fraction; MCS, mechanical circulatory support; NSTEMI, non-ST segment elevation myocardial infarction; NR, not primary reperfused; PCI, percutaneous coronary intervention; pPCI, primary PCI; PE, pulmonary embolism; PAC, pulmonary artery catheterization; PI, pharmacoinvasive strategy; SCAI, Society for Cardiovascular Angiography and Interventions; STEMI, ST segment elevation myocardial infarction; VF, ventricular fibrillation; VT, ventricular tachycardia.

### Non-AMI cardiogenic shock

Women presented at an older age (*P* < 0.001), while men had higher BMI (*P* = 0.012) and smoking prevalence (53% vs. 16.1%, *P* < 0.001). Men also had more dyslipidemia, hypertension, COPD, and prior MI, whereas women more often had a history of atrial fibrillation.

Women showed higher LVEF (54% vs. 40%, *P* < 0.001) but lower hemoglobin (*P* < 0.001) and eGFR (*P* = 0.001). Men had higher BUN, creatinine, and leukocyte counts, while women had higher platelet counts and glucose levels (all *P* < 0.001). AST and ALT were higher in men, LDH was similar, and minimum pH was lower in women (*P* = 0.003), though peak lactate did not differ.

Regarding management, PAC use was more common in men (2.6% vs. 1.3%, *P* = 0.001). Rates of mechanical ventilation and hemodialysis were similar. Vasoactive therapy showed sex-specific patterns: levosimendan (6.1% vs. 2.6%, *P* < 0.001) and dobutamine (*P* < 0.001) were used more often in men, while norepinephrine was more frequent in women (*P* = 0.045). MCS increased in men (1.9% vs. 1.2%, *P* = 0.058). No Impella was used, and ECMO use was rare and balanced (4 men and 6 women, *P* = 0.36).

Outcomes again favored men. Mortality was higher in women (*P* = 0.025), as was AKI (39.6% vs. 35.6%, *P* = 0.007). No significant sex difference was observed in SCAI stage distribution (Table 1).

### Specific risk factors for mortality in CS in women and men

Tables 2 and 3 delineate the differences observed in survivors vs. non-survivors in men and women with AMI-CS and non-AMI-CS. Moreover, Table S4 in the supplementary data represents the univariate hazard ratio (HR) of men and women with AMI-CS and non-AMI-CS with in-hospital mortality.

**Table 2:** Demographics, clinical parameters, management, and outcomes of patients with AMI cardiogenic shock, stratified by sex and survival.


		MEN IN AMI-CS			WOMEN IN AMI-CS		
	
		SURVIVORS (*n* = 3369)	NON-SURVIVORS (*n* = 656)	*P*-VALUE	SURVIVORS (*n* = 747)	NON-SURVIVORS (*n* = 244)	*P*-VALUE

Age (years)		59 (52–67)	65 (57–73)	<0.001	66 (57–74)	70 (63–78)	<0.001

Body mass index (kg/m^2^)		27.31 (24.84–29.76)	26.57 (24.22–28.72)	<0.001	26.89 (23.98–29.9)	25.96 (23.79–29.3)	0.11

Smoking history (%)		2275 (67.5)	433 (66)	0.447	251 (33.6)	69 (28.3)	0.123

Dyslipidemia (%)		1009 (29.9)	192 (29.3)	0.727	232 (31.1)	66 (27)	0.236

Hypertension (%)		1619 (48.1)	356 (54.3)	0.004	525 (70.3)	176 (72.1)	0.581

COPD (%)		50 (1.5)	26 (4)	<0.001	24 (3.2)	9 (3.7)	0.719

Heart failure history (%)		263 (7.8)	86 (13.1)	<0.001	72 (9.2)	35 (14.3)	0.04

Chronic kidney disease (%)		155 (4.6)	84 (12.8)	<0.001	66 (8.8)	25 (10.2)	0.508

Diabetes mellitus (%)		1301 (38.6)	351 (53.3)	<0.001	412 (55.2)	169 (69.3)	<0.001

Previous MI (%)		746 (22.1)	164 (25)	0.11	145 (19.4)	38 (15.6)	0.18

Previous PCI (%)		428 (12.7)	70 (10.7)	0.148	70 (9.4)	9 (3.7)	0.004

Previous CABG (%)		85 (2.5)	17 (2.6)	0.919	26 (3.5)	8 (3.3)	0.88

Stroke history (%)		76 (2.3)	26 (4)	0.011	17 (2.3)	8 (3.3)	0.386

Previous atrial fibrillation (%)		61 (1.8)	15 (2.3)	0.413	24 (3.2)	14 (5.7)	0.075

Type of ACS (%)	NSTEMI	930 (27.6)	156 (23.8)	0.044	304 (40.7)	77 (31.6)	0.011
	
	STEMI	2439 (72.4)	500 (76.2)		443 (59.3)	167 (68.4)	

Admission vital signs and paraclinical work-up							

Systolic blood pressure (mmHg)		128 (110–145)	110 (90–124)	<0.001	128 (110–150)	107 (90–126)	<0.001

Diastolic blood pressure (mmHg)		80 (70–90)	70 (59–80)	<0.001	75 (64–90)	65 (51–79)	<0.001

Medium arterial pressure (mmHg)		94 (83.33–106.67)	80.5 (68.83–93.33)	<0.001	93.33 (80–106.67)	78.83 (66.33–93.33)	<0.001

Heart rate (bpm)		80 (70–95)	90 (72–109)	<0.001	80 (68–95)	88 (67–100)	0.016

Respiratory rate (rpm)		18 (16–20)	20 (18–24)	<0.001	18 (17–21)	20 (18–24)	<0.001

LVEF (%)		47 (37–55)	30 (24–40)	<0.001	50 (40–57)	35 (28–45)	<0.001

Hemoglobin (g/dL)		15.5 (14.1–16.7)	14.4 (12.85–16)	<0.001	13.2 (11.6–14.5)	12.6 (11.3–14)	0.003

Leukocytes (c*10^9^ L)		11.37 (8.97–14.16)	12.9 (9.8–16.7)	<0.001	10.8 (8.42–13.6)	11.8 (9.3–14.6)	0.001

Neutrophils (%)		77.9 (69–84.1)	82 (75–87)	<0.001	77 (67–84)	80 (72.3–85)	0.001

Platelets (c*10^9^ L)		224 (187–267)	212 (164–267)	<0.001	255 (209–309)	242 (173–292)	<0.001

Glucose (mg/dL)		156 (123–225)	192 (135–288.5)	<0.001	178.5 (130–259)	186 (134–287)	0.175

BUN (mg/dL)		18 (14–25)	29 (20–46)	<0.001	21 (15–30)	29.5 (20.8–43)	<0.001

Creatinine (mg/dL)		1.06 (0.9–1.3)	1.6 (1.2–2.4)	<0.001	0.9 (0.7–1.36)	1.3 (0.97–2)	<0.001

eGFR (mL/min/1.73 m^2^)		76.99 (57.67–93.73)	44.26 (26.34–66.86)	<0.001	63.64 (39.67–88.07)	41.48 (25.25–61.64)	<0.001

Chloride (mEq/L)		103 (100–105)	103 (99.55–106)	0.734	102 (99–105)	101 (97.9–105)	0.073

Sodium (mEq/L)		137 (134–139)	136 (134–139)	0.171	136 (133.75–139)	136 (132–139)	0.145

Potassium (mEq/L)		4.2 (3.9–4.6)	4.5 (4–5)	<0.001	4.2 (3.9–4.6)	4.49 (4–5)	<0.001

Albumin (g/dL)		3.8 (3.44–4.07)	3.37 (3–3.72)	<0.001	3.6 (3.29–3.9)	3.3 (3–3.7)	<0.001

AST (UI/L)		76.45 (34–209)	190.5 (56.85–509)	<0.001	53.6 (28.65–149)	133 (48.5–323)	<0.001

ALT (UI/L)		48.9 (30–86)	80 (41.9–186.7)	<0.001	37 (21.3–71)	61.8 (31–158)	<0.001

Lactate dehydrogenase (UI/L)		451.5 (250–848.5)	846 (396–1643)	<0.001	381 (231.5–695.5)	645.5 (399–1117)	<0.001

C-reactive protein (mg/L)		11.8 (3.7–50)	51.7 (12.66–144)	<0.001	12.3 (4.5–46.5)	38 (9.8–120)	<0.001

72h – minimum pH		7.4 (7.36–7.44)	7.31 (7.21–7.38)	<0.001	7.4 (7.36–7.43)	7.32 (7.21–7.4)	<0.001

72h – maximum lactate		2.4 (2–3.1)	3.25 (2–6.26)	<0.001	2.3 (2–3.1)	3.2 (1.9–6.75)	<0.001

Cardiogenic shock management							

Primary reperfusion (%)	NR*	2054 (61)	499 (76.1)	<0.001	523 (70)	194 (79.5)	0.014
	
	pPCI	958 (28.4)	119 (18.1)		166 (22.2)	39 (16.0)	
	
	PI	357 (10.6)	38 (5.8)		58 (7.8)	11 (4.50)	

PAC (%)		140 (4.2)	143 (21.8)	<0.001	36 (4.8)	30 (12.3)	<0.001

Mechanical ventilation (%)		253 (7.5)	414 (63.1)	<0.001	61 (8.2)	125 (51.2)	<0.001

Hemodialysis (%)		54 (1.6)	86 (13.1)	<0.001	24 (3.2)	28 (11.5)	<0.001

Number of vasoactives (%)	0	2639 (78.3)	105 (16)	<0.001	560 (75)	43 (17.6)	<0.001
	
	1	391 (11.6)	98 (14.9)		108 (14.5)	49 (20.1)	
	
	2	200 (5.9)	162 (24.7)		51 (6.8)	62 (25.4)	
	
	3	112 (3.3)	221 (33.7)		19 (2.5)	69 (28.3)	
	
	4	27 (0.8)	70 (10.7)		9 (1.2)	21 (8.60)	

Levosimendan (%)		110 (3.3)	114 (17.4)	<0.001	17 (2.3)	30 (12.3)	<0.001

Dobutamine (%)		419 (12.4)	400 (61)	<0.001	96 (12.9)	132 (54.1)	<0.001

Norepinephrine (%)		560 (16.6)	500 (76.2)	<0.001	151 (20.2)	186 (76.2)	<0.001

Vasopressin (%)		146 (4.3)	351 (53.5)	<0.001	39 (5.2)	116 (47.5)	<0.001

MCS (%)		298 (8.8)	296 (45.1)	<0.001	56 (7.5)	59 (24.2)	<0.001

Cardiogenic shock severity							

SCAI (%)	E	207 (6.1)	360 (54.9)	<0.001	51 (6.8)	123 (50.4)	<0.001
	
	D	243 (7.2)	149 (22.7)		63 (8.4)	49 (20.1)	
	
	C	485 (14.4)	88 (13.4)		130 (17.4)	42 (17.2)	
	
	B	2434 (72.2)	59 (9)		503 (67.3)	30 (12.3)	

Cardiogenic shock outcomes							

Stroke (%)		32 (0.9)	14 (2.1)	0.009	9 (1.2)	11 (4.50)	0.001

VT/VF (%)		242 (7.2)	296 (45.1)	<0.001	39 (5.2)	89 (36.5)	<0.001

GIB (%)		41 (1.2)	52 (7.9)	<0.001	16 (2.1)	14 (5.70)	0.004

PE (%)		0 (0.0)	0	NA	0 (0.0)	1 (0.40)	0.246

AKI (%)		553 (16.4)	328 (50)	<0.001	180 (24.1)	127 (52)	<0.001

Nosocomial pneumonia (%)		105 (3.1)	74 (11.3)	<0.001	19 (2.5)	23 (9.40)	<0.001

Sepsis (%)		69 (2.0)	91 (13.9)	<0.001	15 (2.0)	26 (10.7)	<0.001


*NR patients comprise NSTEMI patients or STEMI late presenters that did not have a primary reperfusion. ACS, acute coronary syndrome; AMI, acute myocardial infarction; AKI, acute kidney injury; ALT, alanine aminotransferase; AST, aspartate aminotransferase; BUN, blood urea nitrogen; CABG, coronary artery bypass grafting; COPD, chronic obstructive pulmonary disease; CRP, C-reactive protein; CS, cardiogenic shock; eGFR, estimated glomerular filtration rate; GIB, gastrointestinal bleeding; LVEF, left ventricular ejection fraction; MCS, mechanical circulatory support; NSTEMI, non-ST segment elevation myocardial infarction; NR, not primary reperfused; PCI, percutaneous coronary intervention; pPCI, primary PCI; PE, pulmonary embolism; PAC, pulmonary artery catheterization; PI, pharmacoinvasive strategy; SCAI, Society for Cardiovascular Angiography and Interventions; STEMI, ST segment elevation myocardial infarction; VF, ventricular fibrillation; VT, ventricular tachycardia.

**Table 3:** Demographics, clinical parameters, management, and outcomes of patients with non-AMI cardiogenic shock, stratified by sex and survival.


		WOMEN IN NON-AMI-CS			MEN IN NON-AMI-CS		
	
		SURVIVORS (*n* = 1446)	NON-SURVIVORS (*n* = 536)	*P*-VALUE	SURVIVORS (*n* = 1846)	NON-SURVIVORS (*n* = 586)	*P*-VALUE

Age (years)		63 (50–75)	63 (50–75)	0.931	60 (46–71)	60 (46–71)	0.968

Body mass index (kg/m^2^)		25.39 (22.64–29.38)	24.78 (22.18–28.35)	0.002	25.78 (23.5–28.48)	25.39 (23.03–27.76)	0.005

Smoking history (%)		229 (15.8)	91 (17.0)	0.054	991 (53.7)	298 (50.9)	0.232

Dyslipidemia (%)		190 (13.1)	63 (11.8)	0.411	388 (21.0)	102 (17.4)	0.058

Hypertension (%)		729 (50.4)	235 (43.8)	0.009	798 (43.2)	241 (41.1)	0.37

COPD (%)		128 (8.90)	73 (13.6)	0.002	99 (5.40)	36 (6.10)	0.472

Heart failure history (%)		554 (38.3)	272 (50.7)	<0.001	953 (51.6)	342 (58.4)	0.004

Chronic kidney disease (%)		170 (11.8)	81 (15.1)	0.046	288 (15.6)	120 (20.5)	0.006

Diabetes mellitus (%)		384 (26.6)	127 (23.7)	0.196	469 (25.4)	131 (22.4)	0.135

Previous MI (%)		107 (7.40)	38 (7.10)	0.814	368 (19.9)	131 (22.4)	0.206

Previous PCI (%)		45 (3.10)	17 (3.20)	0.946	185 (10.0)	60 (10.2)	0.879

Previous CABG (%)		26 (1.80)	9 (1.70)	0.858	91 (4.90)	25 (4.30)	0.512

Stroke history (%)		116 (8.00)	57 (10.6)	0.067	89 (4.80)	44 (7.50)	0.013

Previous atrial fibrillation (%)		427 (29.5)	223 (41.6)	<0.001	331 (17.9)	114 (19.5)	0.406

Admission vital signs and paraclinical work-up							

Systolic blood pressure (mmHg)		110 (90–136)	100 (85–120)	<0.001	110 (90–130)	100 (88–120)	<0.001

Diastolic blood pressure (mmHg)		67 (60–80)	60 (50–72)	<0.001	69 (60–80)	60 (50–72)	<0.001

Medium arterial pressure (mmHg)		83.33 (70–96.67)	74.5 (63.33–89.83)	<0.001	83.33 (70–95)	73.33 (64–89.33)	<0.001

Heart rate (bpm)		88 (60–110)	98 (77–115)	<0.001	89 (66–109)	94 (77–110)	<0.001

Respiratory rate (rpm)		20 (18–24)	22 (18–26)	<0.001	20 (18–24)	22 (18–26)	<0.001

LVEF (%)		55 (40–60)	50 (32–60)	<0.001	43 (26–58)	35 (23–55)	<0.001

Hemoglobin (g/dL)		12.7 (10.7–14.3)	12.5 (10.1–14.3)	0.178	14.2 (12.3–15.7)	13.63 (11.2–15.5)	<0.001

Leukocytes (c*10^9^ L)		9.19 (6.83–12.84)	10.45 (7.2–14.9)	<0.001	9.5 (7.47–12.6)	10.9 (7.7–14.9)	<0.001

Neutrophils (%)		76.65 (67.7–85)	83 (74.6–88.8)	<0.001	76.5 (68–84)	82 (75–88.65)	<0.001

Platelets (c*10^9^ L)		206 (155–270)	163 (112–233)	<0.001	194 (150–248)	164.5 (122–220)	<0.001

Glucose (mg/dL)		125 (101–168)	128.5 (101–171)	0.551	120 (100–154)	118 (96–165)	0.418

BUN (mg/dL)		26 (16.8–44)	39 (23–64)	<0.001	29 (19–48)	42 (26–65)	<0.001

Creatinine (mg/dL)		1.1 (0.8–1.8)	1.6 (1.1–2.65)	<0.001	1.4 (1–2)	1.8 (1.3–2.9)	<0.001

eGFR (mL/min/1.73 m^2^)		53.71 (29.67–79.26)	34.3 (18.66–59)	<0.001	56.17 (33.5–81.21)	39.14 (21.41–62.84)	<0.001

Chloride (mEq/L)		101 (97–105)	99.35 (94–104)	<0.001	101.46 (97–105)	99 (94–103)	<0.001

Sodium (mEq/L)		136 (131.42–138.6)	134 (130–138)	<0.001	136 (132–139)	134 (129–137)	<0.001

Potassium (mEq/L)		4.31 (3.9–5)	4.6 (4.08–5.4)	<0.001	4.4 (4–5)	4.6 (4.1–5.3)	<0.001

Albumin (g/dL)		3.44 (3.02–3.8)	3.16 (2.66–3.6)	<0.001	3.49 (3.02–3.87)	3.15 (2.62–3.6)	<0.001

AST (UI/L)		32 (21.09–53.5)	48 (29.7–104.4)	<0.001	33 (22–68)	55 (30.13–141)	<0.001

ALT (UI/L)		26 (16.4–48)	32 (19–72)	<0.001	31 (18.7–69.4)	39 (22.6–106)	<0.001

Lactate dehydrogenase (UI/L)		311.5 (204.4–499)	499.5 (301–893.5)	<0.001	310 (202–528)	446 (272–771)	<0.001

C-reactive protein (mg/L)		17.3 (5.86–54)	51 (16–120)	<0.001	22 (7.4–70.3)	58.3 (20–130)	<0.001

72h – minimum pH		7.39 (7.34–7.44)	7.31 (7.19–7.4)	<0.001	7.4 (7.35–7.44)	7.34 (7.21–7.41)	<0.001

72h – maximum lactate		2.3 (1.8–3.1)	3.3 (2.1–6.35)	<0.001	2.3 (1.9–3.2)	3.2 (2–5.9)	<0.001

Cardiogenic shock management							

PAC (%)		14 (1.00)	11 (2.10)	0.055	35 (1.90)	29 (4.90)	<0.001

Mechanical ventilation (%)		166 (11.5)	251 (46.8)	<0.001	190 (10.3)	292 (49.8)	<0.001

Hemodialysis (%)		66 (4.60)	53 (9.90)	<0.001	78 (4.20)	79 (13.5)	<0.001

Number of vasoactives (%)	0	1025 (70.9)	133 (24.8)	<0.001	1265 (68.5)	125 (21.3)	<0.001
	
	1	289 (20.0)	128 (23.9)		357 (19.3)	141 (24.1)	
	
	2	105 (7.30)	178 (33.2)		167 (9.00)	182 (31.1)	
	
	3	22 (1.50)	88 (16.4)		45 (2.40)	117 (20.0)	
	
	4	5 (0.30)	9 (1.70)		12 (0.70)	21 (3.60)	

Levosimendan (%)		29 (2.00)	22 (4.10)	0.009	98 (5.30)	51 (8.70)	0.003

Dobutamine (%)		164 (11.3)	148 (27.6)	<0.001	326 (17.7)	222 (37.9)	<0.001

Norepinephrine (%)		316 (21.9)	378 (70.5)	<0.001	364 (19.7)	418 (71.3)	<0.001

Vasopressin (%)		76 (5.30)	236 (44.0)	<0.001	86 (4.70)	249 (42.5)	<0.001

MCS (%)		19 (1.30)	8 (1.50)	0.761	36 (2.00)	14 (2.40)	0.514

Cardiogenic shock severity							

SCAI (%)	E	137 (9.50)	234 (43.7)	<0.001	189 (10.2)	244 (41.6)	<0.001
	
	D	111 (7.70)	107 (20.0)		181 (9.80)	131 (22.4)	
	
	C	327 (22.6)	101 (18.8)		402 (21.8)	120 (20.5)	
	
	B	871 (60.2)	94 (17.5)		1074 (58.2)	91 (15.5)	

Cardiogenic shock outcomes							

Stroke (%)		14 (1.00)	15 (2.80)	0.003	15 (0.80)	16 (2.70)	<0.001

VT/VF (%)		60 (4.10)	120 (22.4)	<0.001	85 (4.60)	152 (25.9)	<0.001

GIB (%)		16 (1.10)	29 (5.40)	<0.001	26 (1.40)	23 (3.90)	<0.001

PE (%)		5 (0.30)	2 (0.40)	1.0	4 (0.20)	4 (0.70)	0.101

AKI (%)		501 (34.6)	283 (52.8)	<0.001	584 (31.6)	282 (48.1)	<0.001

Nosocomial pneumonia (%)		43 (3.00)	31 (5.80)	0.003	41 (2.20)	41 (7.00)	<0.001

Sepsis (%)		50 (3.50)	104 (19.4)	<0.001	67 (3.60)	125 (21.3)	<0.001


ACS, acute coronary syndrome; AMI, acute myocardial infarction; AKI, acute kidney injury; ALT, alanine aminotransferase; AST, aspartate aminotransferase; BUN, blood urea nitrogen; CABG, coronary artery bypass grafting; COPD, chronic obstructive pulmonary disease; CRP, C-reactive protein; CS, cardiogenic shock; eGFR, estimated glomerular filtration rate; GIB, gastrointestinal bleeding; LVEF, left ventricular ejection fraction; MCS, mechanical circulatory support; NSTEMI, non-ST segment elevation myocardial infarction; NR, not primary reperfused; PCI, percutaneous coronary intervention; pPCI, primary PCI; PE, pulmonary embolism; PAC, pulmonary artery catheterization; PI, pharmacoinvasive strategy; SCAI, Society for Cardiovascular Angiography and Interventions; STEMI, ST segment elevation myocardial infarction; VF, ventricular fibrillation; VT, ventricular tachycardia.

### Specific risk factors in the multivariate analysis of mortality in AMI-CS

In men with AMI-CS, type 2 diabetes (*P* = 0.003) and STEMI presentation (*P* = 0.004) were independently associated with higher mortality. Primary PCI was protective, whereas pharmacoinvasive and late/non-reperfusion strategies were not. PAC use was associated with lower mortality (HR 0.75, 95% CI 0.59–0.96, *P* = 0.023). Mechanical ventilation markedly increased the risk. Mortality rose progressively with higher SCAI-CSWG: HRs were 6.29 (95% CI 4.06–9.75), 4.07 (2.66–6.23), and 3.22 (2.12–4.90) for stages E, D, and C, respectively (all *P* < 0.001 vs. stage B). Additional predictors included older age, reduced LVEF, higher BUN, and elevated lactate. Albumin showed an inverse association with mortality (HR 1.01, 95% CI 1.01–1.10, *P* = 0.002).

In women with AMI-CS, mechanical ventilation was strongly associated with increased mortality (HR 1.76, 95% CI 1.20–2.58, *P* = 0.004). Higher SCAI-CSWG stage conferred a stepwise increase in risk compared with stage B: HRs were 4.90 (95% CI 2.75–8.72), 3.02 (1.63–5.59), and 2.86 (1.62–5.04) for stages E, D, and C, respectively (all *P* < 0.001). Additional predictors included older age, reduced LVEF, and elevated lactate.

### Specific risk factors in the multivariate analysis of mortality in non-AMI-CS

**Figure 2:**
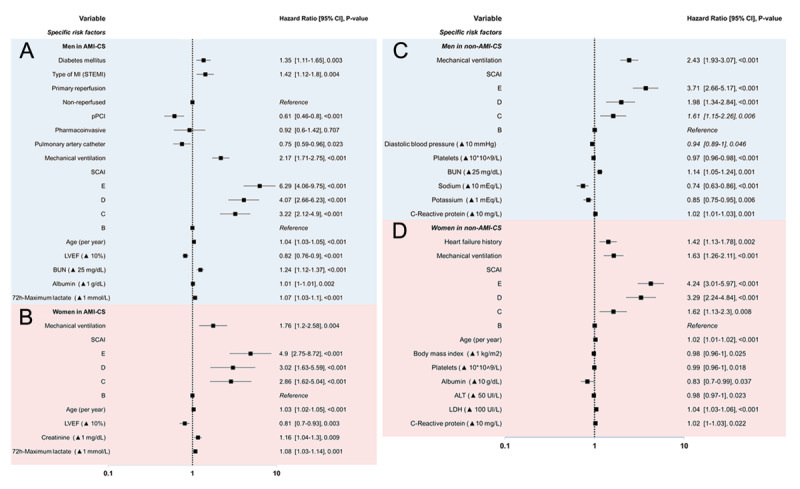
Cox Forrest of multivariate regression of sex-specific factors related to mortality in AMI-CS in men **(A)** and women **(B)**; and in non-AMI-CS men **(C)** and women **(D)**. Blue shade = men, red shade = women.

In men with non-AMI-CS, higher SCAI-CSWG stages predicted mortality, with HRs of 3.71 (95% CI 2.66–5.17, *P* < 0.001) for stage E, 1.98 (1.34–2.84, *P* < 0.001) for D, and 1.61 (1.15–2.26, *P* = 0.006) for C compared with stage B. Mechanical ventilation and lower diastolic blood pressure were also associated with increased risk. Additional predictors included lower platelet counts, higher BUN and CRP, while higher sodium and potassium levels were protective (Figure 2 and Graphical Abstract).

In women with non-AMI-CS, prior heart failure and mechanical ventilation were independently associated with increased mortality. Higher SCAI-CSWG stages conferred a stepwise rise in risk: HRs were 4.24 (95% CI 3.01–5.97, *P* < 0.001) for stage E, 3.29 (2.24–4.84, *P* < 0.001) for D, and 1.62 (1.13–2.30, *P* = 0.008) for C compared with stage B. Additional predictors included older age, higher albumin, ALT, LDH, and CRP, whereas higher BMI was protective (Figure 2 and Graphical Abstract).

### Treatment intensity in AMI and non-AMI-CS

In AMI-CS treatment intensity varied across SCAI-CSWG stages with consistent gender differences. Invasive procedures such as PAC and hemodialysis were used less frequently in women, particularly in stage E. Mechanical circulatory support – especially IABP – was more often used in men across stages D and E. Among vasoactives, norepinephrine and levosimendan were more common in men (notably stage D), whereas vasopressin use was higher in women (Table S3).

In non-AMI-CS, gender-related treatment patterns were subtler. PAC was more prevalent in men, especially in stage D, as was hemodialysis. Rates of MCS did not differ between sexes across SCAI stages. In contrast, vasoactive use diverged: norepinephrine and vasopressin were more common in women (stages C and D), while men more often received dobutamine and levosimendan across stages C–E (Table S4).

### Sex-based differences in mortality and survival (AMI-CS and non–AMI-CS)

In AMI-CS, women had shorter survival (Log-rank *P <* 0.001), 30-day RMST of 2.11 (1.12–3.08, *P <* 0.001) days fewer of survival with a HR of 1.48 (1.28–1.72, *P <* 0.001) and adjusted HR of 1.24 (1.05–1.46; *P* = 0.011).

**Figure 3:**
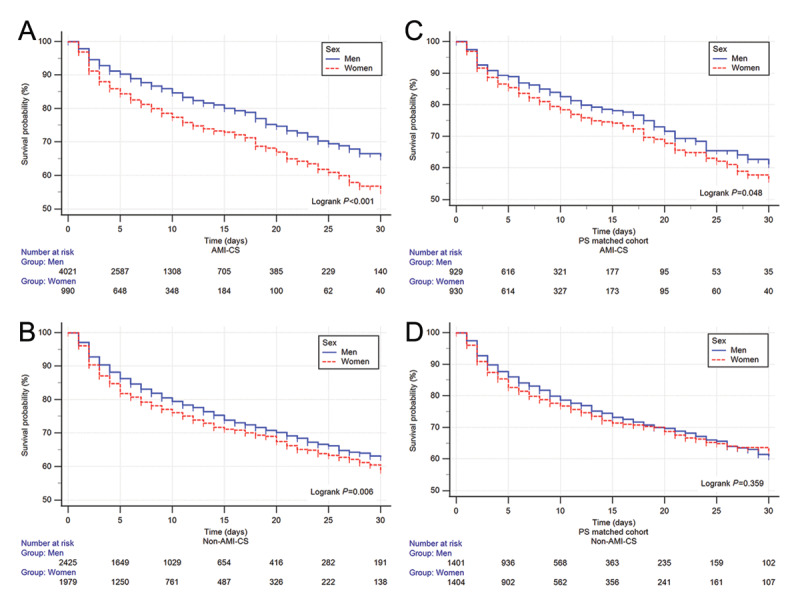
Kaplan–Meir curves of Acute myocardial infarction (AMI) related cardiogenic shock (CS) in the whole cohort (A) and non-AMI-CS (B) in the whole cohort. The propensity score matched the AMI-CS (C) and non-AMI-CS (D) cohorts. Blue = men, red = women.

**Figure 4:**
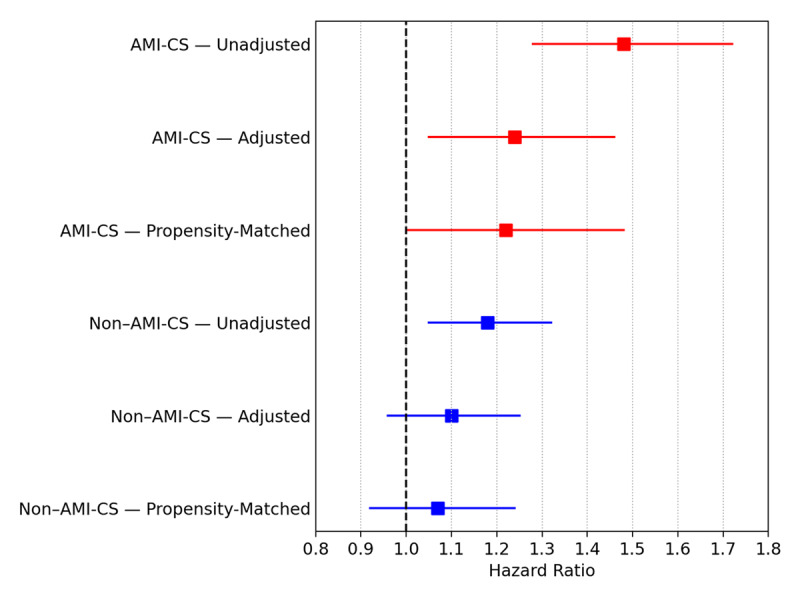
Forest plot of sex-based differences in mortality in AMI-CS (acute myocardial infarction–related cardiogenic shock, red) and non–AMI-CS (blue), showing hazard ratios (HR) with 95% confidence intervals (CI) for women versus men across unadjusted, adjusted, and propensity score–matched (PSM) models.

For non-AMI-CS survival was also reduced in women (Log-rank *P =* 0.006), a 30-day RMST, a difference of diminished survival of 0.85 (0.08–1.62, *P =* 0.03) days, a HR of 1.18 (1.05–1.32; *P =* 0.007), and a non-significant adjusted HR of 1.10 (0.96–1.25, *P* = 0.175; Figures 3 and 4).

### Propensity score matching sex-based differences in mortality and survival (AMI-CS and non–AMI-CS)

After propensity score matching, sex-related differences in survival were attenuated. In AMI-CS (931 vs. 931), women showed a borderline difference by Log-rank testing (*P* = 0.048), but no significant differences were observed in 30-day RMST (1.09 days, 95% CI –0.17 to 2.35; *P* = 0.09) or mortality (HR 1.22, 95% CI 1.00–1.48; *P* = 0.052). In non-AMI-CS (1406 vs. 1406), no differences were identified in Log-rank *P* = 0.359, 30-day RMST 0.4 (–0.55–1.36, *P =* 0.407), or HR 1.07 (0.92–1.24, *P =* 0.366; Figures 3 and 4, full analysis supplementary Figures S3 and Table S6).

## Discussion

In this study, we conducted a comprehensive analysis of sex-specific disparities in CS within a large Latin-American cohort. Women have unique risk factors impacting outcomes more than sex itself in AMI-CS and non-AMI-CS. Our findings highlight notable differences in demographic, clinical, and hemodynamic profiles between male and female CS patients across etiologies. The analysis showed risk factor differences between male and female survivors and non-survivors, though some are interconnected, underscoring the complexity of CS prognosis. When calculating the propensity score match, mortality differences disappeared, suggesting women have higher comorbidities and lower treatment intensity than men, leading to higher observed mortality.

### AMI-CS sex differences

In AMI-CS, women presented at an older age with more hypertension and diabetes, as in CSWG and CUPLRIT-SHOCK analysis (9, 14), in contrast to our study, since men showed a higher median BMI and smoking prevalence in this Latin cohort. Women also exhibited lower hemoglobin, lower eGFR, and higher hemo-metabolic compromise, reflected by lower 72 h-minimum pH – findings only partially captured in prior registries (CSWG, INOVA) (7,9).

In the realm of therapeutic interventions, sex-specific disparities were evident. Women received significantly less primary reperfusion than men, contrasting with the INOVA registry (no differences, *P* = 0.32) and the NIS registry (66.7% vs. 65.7%, *P* < 0.001), but aligning with CULPRIT-SHOCK, where women trended toward lower reperfusion rates (7,14,15).

Regarding advanced therapies, women with AMI-CS were less likely to receive MCS and more likely to be treated with vasopressors, while no differences emerged in non-AMI-CS. Yan et al. similarly reported reduced use of LVADs (OR 0.78, 0.64–0.94) and greater vasopressor exposure (OR 1.26, 1.05–1.5) in women, without mortality differences (7,16).

In contrast, INOVA-SHOCK showed no sex differences, and non-AMI-CS cohorts even demonstrated higher MCS and vasopressor use in women (7).

Adverse outcomes also showed sex-specific differences. Women experienced higher rates of stroke and AKI, and prior work by Vallabhajosyula et al. demonstrated that female patients with AKI were less likely to receive advanced therapies such as MCS, mechanical ventilation, or hemodialysis, despite greater comorbidity and higher mortality (19). Consistently, in our cohort, women presented more often with advanced SCAI-CSWG (C–E) and higher unadjusted mortality, findings that contrast with the CSWG report, where admission stage distribution and outcomes were similar between sexes (9).

### Non-AMI-CS sex differences

In non-AMI CS, we observed distinct sex-related patterns. Similar to CSWG and Sundermeyer et al. (9,18), men were younger and more likely to have prior MI, whereas women were older with a greater prevalence of atrial fibrillation and lower eGFR despite lower creatinine levels. Significantly, in our cohort, age independently predicted outcomes only in women, suggesting heightened vulnerability of older females with non-AMI CS. Echocardiographic assessment also showed higher LVEF in women, consistent with prior non-ischemic shock registries (18).

Consistent with Sundermeyer, creatinine was higher in men; we observed the same pattern. Although eGFR was not reported in CSWG or Sundermeyer, in our cohort, men had higher creatinine, but women had lower eGFR by the CKD-EPI formula. Hepatic enzymes also differed by sex: AST and ALT were higher in men, contrasting with CSWG, where ALT did not differ (women 290 vs. men 266 U/L; *P* = 0.07), and AST was not reported. Finally, women had lower arterial pH despite similar peak lactate, suggesting a sex-specific pathway to metabolic acidosis not captured by lactate; prior registries reported no sex differences in lactate or pH (9,18).

Regarding hemodynamic support, PAC use was very low overall; this contrasts sharply with CSWG, where nearly half of both sexes underwent PAC, reflecting regional differences and the legacy indication of PAC mainly for AMI-CS. Mechanical ventilation and vasoactive requirements showed no sex differences, in line with CSWG and Sundermeyer (9,18). The SCAI-CSWG classification demonstrated no sex disparity, in contrast to the CSWG registry, in which women appeared to have higher stages (9).

### Specific sex risk factors

In AMI-CS, women exhibit a heightened reliance on creatinine levels as a prognostic marker, underscoring the importance of renal function, and possibly related to a lower intensity of therapies in this population (17). Conversely, men demonstrate a propensity toward classical risk factors such as DM2, type of MI, and the presence of primary reperfusion interventions.

Furthermore, PAC may refine risk stratification and guide therapy; however, its effect must be interpreted cautiously, given the lower use observed in non-AMI-CS and women with AMI-CS. Previously, our group demonstrated that PAC, as a guidance to decongestion, could serve as a goal in AMI-CS (19,20) while Kanwar et al. reported that early PAC use in non-AMI-CS reduced mortality (19,20).

Despite these sex-specific disparities, areas of convergence are evident, with age, SCAI-CSWG stage, LVEF, and lactate levels representing common denominators in risk assessment across both sexes.

In non-AMI-CS, convergent risk factors were: mechanical ventilation, SCAI-CSWG stages, platelets, and C-reactive protein. Nevertheless, women had more risk factors for the prediction of in-hospital mortality, such as age, BMI, and previous history of heart failure. While the CSWG registry reported higher BMI as associated with increased mortality in non-AMI-CS (21), our findings suggest a paradoxical protective effect of BMI in Latin American women. Also, our study is the first to highlight the prognostic relevance of liver function specifically in women, as albumin, ALT, and LDH emerged as independent predictors. Conversely, men showed a distinctive profile dominated by renal predictors: sodium, potassium, BUN, and diastolic blood pressure.

### Treatment intensity and propensity scores matching insights

A notable sex gap has emerged in the intensity of treatment for AMI-CS. Across various SCAI stages, women consistently receive less aggressive treatment, such as PAC and hemodialysis, which are notably lower in women across SCAI E, contrasting with the CSWG experience (9,22). Given the established prognostic value of PAC in CS (22,23,24), these disparities are unlikely to reflect biological differences and may represent treatment bias.

MCS use, particularly IABP, was also significantly higher in men in SCAI-CSWG D/E. This mirrors prior studies showing underutilization of MCS in women, often attributed to higher vascular complication risk and smaller vessel anatomy (6,9,15,16,25). Notably, our data provide additional insight into vasoactive selection: men were more frequently treated with norepinephrine and levosimendan, while women received vasopressin more often – a pattern not previously reported in AMI-CS.

In the non-AMI-CS, men exhibit a higher prevalence of PAC usage, especially in SCAI-CSWG-D, although MCS use was similar across stages, contrasting with the CSWG cohort, where men received more MCS in HF-CS (9). Women receive higher numbers of vasopressors (norepinephrine and vasopressin), whereas men were prescribed more inotropic (dobutamine and levosimendan); these differences, to our understanding, have not been revealed in previous studies (26).

## Limitations

While our study provides valuable insights into the sex-specific characteristics and outcomes of patients with AMI-CS and non-AMI-CS, several limitations must be acknowledged. First, our retrospective analysis utilized data from a coronary care unit database, which inherently introduces selection bias and limits the ability to establish causality. Additionally, the study was conducted at a single academic tertiary center in Mexico City, potentially limiting the generalization of our findings to other settings and populations. Heterogeneity in the management of CS over time and patient selection for temporary MCS, PAC, or vasoactives evidence operator-specific preferences, further complicating the analysis. This has led to an inherent sex bias treatment in our center and probably among Latin-American women, highlighting a lower treatment intensity in this vulnerable population.

Propensity score matching was employed to create sex-specific cohorts with balanced characteristics. Despite the successful balancing, the observational nature of the registry means that not all potential confounders could be addressed. Finally, our study focused on a specific region (Latin America) where resources and technology constraints may differ from other parts of the world, limiting the generalizability of our findings to other populations with different healthcare systems and resource availability. Despite these limitations, our study provides important insights into the Latino sex-specific characteristics and outcomes of patients with CS, highlighting areas for further research and clinical intervention in this neglected field.

## Conclusion

Our research has revealed that there are distinct sex-related risk factors associated with different types of CS. These findings highlight the importance of moving beyond a ‘sex as a variable’ perspective toward a sex-specific risk profile approach in CS. By recognizing and addressing these sex-specific factors, healthcare providers can optimize treatment strategies and improve outcomes for all patients with CS. Importantly, acknowledging the persistent sex gap in invasive monitoring and advanced therapies underscores the urgent need to mitigate sex bias in practice patterns and to explore its long-term consequences, which could lead to better outcomes, particularly among female patients.

## Diversity and Inclusion

This study focuses on women with CS, and many of our authors are women: A.A.M., MD, is the head of the department at the Coronary Care Unit; A.A.S., MD, is the head of the department at the Heart Failure Clinic; P.R.R. and M.C.L.R., MDs, are attendings at the adult cardiology department. Finally, B.A.D.H. and M.N.P.Q, MDs, are our young researchers at the Coronary Care Unit.

Also, the study focuses on dissecting the data in AMI-CS and non-AMI-CS related data in a particular part of the world, where scarce data (Latin-American) and resources and technology constraints are well known.

## Data Accessibility Statement

The data presented in this study are available on request from the corresponding author.

## Additional File

The additional file for this article can be found as follows:

10.5334/gh.1469.s1 Supplementary file. Tables s1 to s6 and Figure s1 to s3.

## Abbreviations

AKI: Acute kidney injury.

ALT: Alanine transaminase.

AMI-CS: Acute myocardial infarction-related cardiogenic shock.

AST: Aspartate transaminase.

BMI: Body mass index.

COPD: Chronic obstructive pulmonary disease.

CRP: C-reactive protein.

SCAI-CSWG: Society for Cardiac Angiography and Interventions-Cardiogenic Shock Working Group stages.

eGFR: Estimated glomerular filtration rate.

IABP: Intra-aortic balloon pump.

LVEF: Left ventricular ejection fraction.

MCS: Mechanical circulatory support.

NR: Non-early reperfusion.

PAC: Pulmonary artery catheter.

PI: Pharmacoinvasive strategy.

pPCI: Primary percutaneous coronary intervention.

RMST: Restricted mean survival time.
