# Supplementary material for: Sex Disparities in Cardiogenic Shock: Risk Factors, Treatment Intensity, and Mortality in a Single Latin American Country
Source: Glob Heart. 2025 Sep 9;20(1):78. doi: 10.5334/gh.1469 (PMC12427619; doi:10.5334/gh.1469)
Supplement: Supplementary file. — Tables s1 to s6 and Figure s1 to s3. [file gh-20-1-1469-s1.pdf]

Supplementary

Sex Disparities in Cardiogenic Shock: Risk Factors, Treatment Intensity, and Mortality in a Single Latin American Country

Content

Methods 2

Table S1. Etiologies of Non-Acute Myocardial Infarction Cardiogenic Shock (non-AMI-CS) in Men and Women 3

Figure S1. Etiologies of Non-Acute Myocardial Infarction Cardiogenic Shock (non-AMI-CS) in Men. 3

Table S2. Missingness of data by AMI Cardiogenic Shock, Stratified by Gender 5

References 5

Table S3. Management Strategies in Patients with AMI Cardiogenic Shock, Stratified by SCAI and Gender 6

Table S4. Management Strategies in Patients with Non-AMI Cardiogenic Shock, Stratified by SCAI and Gender 7

Table S5: Univariate Hazard Ratios for Various Clinical Parameters in Patients with AMI and non-AMI Complicated by CS by Sex 8

Table S6. Demographics, Clinical Parameters, Management, and Outcomes of Patients with AMI and non-AMI Cardiogenic Shock, Stratified by Sex in the Propensity Score Matched Cohort. 12

Methods

We analyzed a coronary care unit database (n=28,054) from late 2015 to September 2023 at the Instituto Nacional de Cardiología Ignacio Chavez, which is an academic tertiary and reference center in Mexico City with a focus on only cardiovascular diseases from which we include patients with the diagnosis of CS was defined as Society for Cardiac Angiography and Interventions-Cardiogenic Shock Working Group (SCAI-CSWG) score.[1], a sustained episode of at least 1 of the following: systolic blood pressure (SBP) <90 mmHg for at least 30 minutes, use of vasoactive agents to maintain SBP, cardiac index <2.2 L/min/m2 in the absence of hypovolemia, each determined to be secondary to cardiac dysfunction, or use of an MCS device for clinically suspected CS, and include those in SCAI B or higher maximum score at the first 72- h. No additional exclusion criteria were applied, except for patients without a confirmed diagnosis of cardiogenic shock.

Patient demographic, laboratory, and hemodynamic data were collected at a single time point as close to admission as possible and across hospitalization at the time. Data was collected during the hospital stay: vasopressor/inotrope administration, MCS placement, pulmonary artery catheter placement, and use of mechanical ventilation. Treatment of CS was left to the primary physician's discretion and not guided by a prescribed algorithm. The hemodialysis was collected for patients with new needs or those requiring chronic renal replacement therapy. Furthermore, (NR) patients comprise NSTEMI patients or STEMI late presenters who did not have a primary reperfusion. AMI-CS was attributed to those whose cause of CS was an acute coronary syndrome per the fourth universal definition of MI,[2] and non-AMI-CS and etiology were attributed by the treating physician (Critical care cardiologist) at the site. (Table S1 and Figure S1-2)

Table S1. Etiologies of Non-Acute Myocardial Infarction Cardiogenic Shock (non-AMI-CS) in Men and Women

	non-AMI-CS	
**Etiology**	**Men (n=2432)**	**Women (n=1982)**
**Others**	136 (5.59)	99 (4.99)
**Ischemic heart disease**	580 (23.85)	177 (8.93)
**Valvular**	623 (25.62)	742 (37.44)
**Cardiomyopathies**	490 (20.15)	341 (17.20)
**Pericardial disease**	35 (1.44)	41 (2.07)
**Heart tumors**	8 (0.33)	30 (1.51)
**Aortic disease**	122 (5.02)	44 (2.22)
**Rhythm/conduction disorders**	243 (9.99)	271 (13.67)
**Cardio-pulmonary**	113 (4.65)	153 (7.72)
**Hypertensive crisis**	8 (0.33)	15 (0.76)
**Congenital heart disease**	74 (3.04)	69 (3.48)

Figure S1. Etiologies of Non-Acute Myocardial Infarction Cardiogenic Shock (non-AMI-CS) in Men.

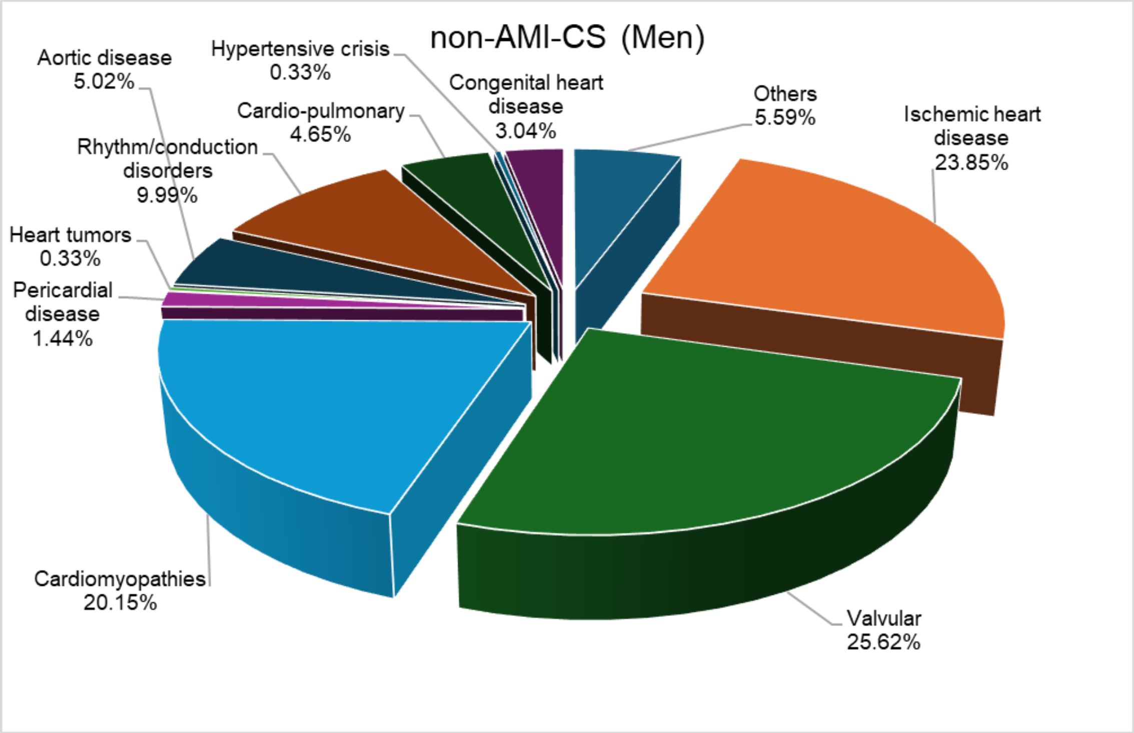

Figure S2. Etiologies of Non-Acute Myocardial Infarction Cardiogenic Shock (non-AMI-CS) in Women.

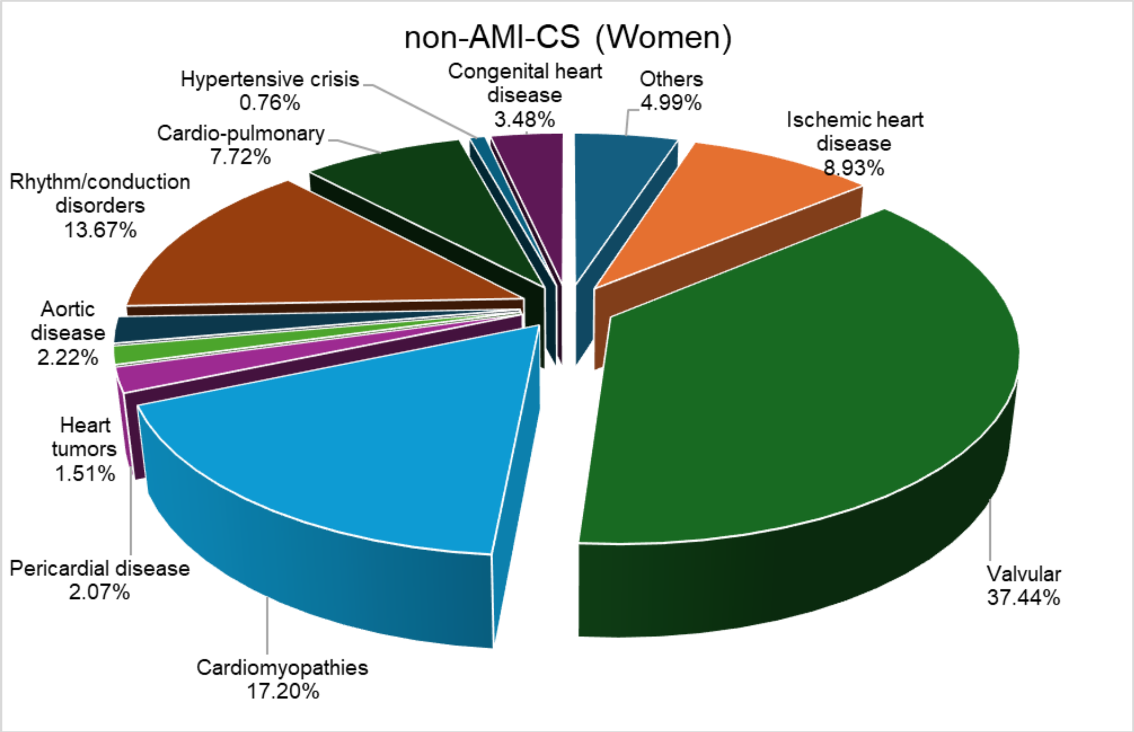

Multivariate analysis and propensity score data were determined as complete data, and no imputation was performed. Table S2, based on the full-length Table 1, provides data for the missingness; if the variable is not presented, the missingness is 0. The normality of the data was assessed for the variables included in the analysis using Kolmogorov-Smirnov, and non-parametric statistical methods were employed for comparisons and analyses as appropriate.

Table S2. Missingness of data by AMI Cardiogenic Shock, Stratified by Gender

	AMI-CS		non-AMI-CS	
	**Men (n=4025)**	**Women (n=991)**	**Men (n=2432)**	**Women (n=1982)**
**Body mass index (%)**	7 (0.17)	3 (0.3)	2 (0.08)	2 (0.1)
**Admission vital signs and paraclinical work-up**				
**Respiratory rate (%)**	0	1 (0.1)	3 (0.12)	1 (0.05)
**LVEF (%)**	222 (5.52)	61 (6.16)	154 (6.33)	142 (7.16)
**Hemoglobin (%)**	16 (0.4)	7 (0.71)	20 (0.82)	9 (0.45)
**Leukocytes (%)**	8 (0.2)	3 (0.3)	8 (0.33)	6 (0.3)
**Neutrophils (%)**	40 (0.99)	11 (1.11)	34 (1.4)	34 (1.72)
**Platelets (%)**	14 (0.35)	5 (0.5)	19 (0.78)	11 (0.55)
**Glucose (%)**	40 (0.99)	8 (0.81)	25 (1.03)	24 (1.21)
**BUN (%)**	39 (0.97)	13 (1.31)	15 (0.62)	28 (1.41)
**Creatinine (%)**	25 (0.62)	6 (0.61)	12 (0.49)	14 (0.71)
**eGFR (%)**	25 (0.62)	6 (0.61)	12 (0.49)	14 (0.71)
**Chloride (%)**	56 (1.39)	24 (2.42)	46 (1.89)	30 (1.51)
**Sodium (%)**	31 (0.77)	9 (0.91)	19 (0.78)	16 (0.81)
**Potassium (%)**	17 (0.42)	4 (0.4)	15 (0.62)	9 (0.45)
**Albumin (%)**	269 (6.68)	88 (8.88)	213 (8.76)	209 (10.54)
**AST (%)**	127 (3.16)	27 (2.72)	128 (5.26)	120 (6.05)
**ALT (%)**	309 (7.68)	88 (8.88)	239 (9.83)	217 (10.95)
**Lactate dehydrogenase (%)**	177 (4.4)	47 (4.74)	192 (7.89)	168 (8.48)
**C-Reactive protein (%)**	248 (6.16)	76 (7.67)	236 (9.7)	243 (12.26)
**72h- Minimum pH (%)**	132 (3.28)	30 (3.03)	88 (3.62)	70 (3.53)

References

[1] Kapur NK, Kanwar M, Sinha SS, Thayer KL, Garan AR, Hernandez-Montfort J, et al. Criteria for Defining Stages of Cardiogenic Shock Severity. J Am Coll Cardiol 2022;80:185–98. https://doi.org/10.1016/J.JACC.2022.04.049.

[2] Thygesen K, Alpert JS, Jaffe AS, Chaitman BR, Bax JJ, Morrow DA, et al. Fourth Universal Definition of Myocardial Infarction (2018). Circulation 2018;138:e618–51. https://doi.org/10.1161/CIR.0000000000000617.

Table S3. Management Strategies in Patients with AMI Cardiogenic Shock, Stratified by SCAI and Gender

AMI-CS		SCAI E	SCAI E	P-value	SCAI D	SCAI D	P-value	SCAI C	SCAI C	P-value	SCAI B	SCAI B	P-value
		Men	Women		Men	Women		Men	Women		Men	Women	
Primary reperfusion (%)	NR*	400 (70.5)	126 (72.4)	0.865	280 (71.4)	85 (75.9)	0.557	414 (72.3)	127 (73.8)	0.896	1459 (58.5)	379 (71.1)	<0.001
	pPCI	135 (23.8)	38 (21.8)		90 (23.0)	23 (20.5)		103 (18.0)	30 (17.4)		749 (30.0)	114 (21.4)	
	PI	32 (5.60)	10 (5.70)		22 (5.60)	4 (3.60)		56 (9.80)	15 (8.70)		285 (11.4)	40 (7.50)	
Mechanical ventilation (%)		386 (68.1)	106 (60.9)	0.08	169 (43.1)	49 (43.8)	0.904	94 (16.4)	24 (14.0)	0.44	18 (0.70)	7 (1.30)	0.185
PAC (%)		180 (31.7)	39 (22.4)	0.018	71 (18.1)	21 (18.8)	0.878	29 (5.10)	4 (2.30)	0.143	3 (0.10)	2 (0.40)	0.215
Hemodialysis (%)		81 (14.3)	21 (12.1)	0.458	28 (7.10)	16 (14.3)	0.018	11 (1.90)	7 (4.10)	0.151	20 (0.80)	8 (1.50)	0.135
MCS (%)		284 (50.1)	54 (31.0)	<0.001	165 (42.1)	21 (18.8)	<0.001	145 (25.3)	40 (23.3)	0.585	0 (0.00)	0 (0.00)	NA
Number of vasoactives (%)	0	46 (8.10)	13 (7.50)	0.059	15 (3.80)	3 (2.70)	0.733	190 (33.2)	54 (31.4)	0.711	2493 (100)	533 (100)	NA
	1	36 (6.30)	22 (12.6)		70 (17.9)	17 (15.2)		383 (66.8)	118 (68.6)		0 (0.00)	0 (0.00)	
	2	55 (9.70)	21 (12.1)		307 (78.3)	92 (82.1)		0 (0.00)	0 (0.00)		0 (0.00)	0 (0.00)	
	3	333 (58.7)	88 (50.6)		0 (0.00)	0 (0.00)		0 (0.00)	0 (0.00)		0 (0.00)	0 (0.00)	
	4	97 (17.1)	30 (17.2)		0 (0.00)	0 (0.00)		0 (0.00)	0 (0.00)		0 (0.00)	0 (0.00)	
Levosimendan (%)		168 (29.6)	40 (23.0)	0.088	48 (12.2)	5 (4.50)	0.018	8 (1.40)	2 (1.20)	1.00	0 (0.00)	0 (0.00)	NA
Dobutamine (%)		448 (79.0)	132 (75.9)	0.378	242 (61.7)	62 (55.4)	0.224	129 (22.5)	34 (19.8)	0.445	0 (0.00)	0 (0.00)	NA
Norepinephrine (%)		506 (89.2)	156 (89.7)	0.877	312 (79.6)	100 (89.3)	0.019	242 (42.2)	81 (47.1)	0.259	0 (0.00)	0 (0.00)	NA
Vasopressin (%)		411 (72.5)	120 (69.0)	0.367	82 (20.9)	34 (30.4)	0.036	4 (0.70)	1 (0.60)	1.00	0 (0.00)	0 (0.00)	NA

*NR patients comprise NSTEMI patients or STEMI late presenters who did not have a primary reperfusion

AMI: Acute Myocardial Infarction, CS: Cardiogenic Shock, PI: pharmacoinvasive strategy, SCAI: Society for Cardiovascular Angiography and Interventions, NR: Not primary reperfused, pPCI: Primary Percutaneous Coronary Intervention, PI: Pharmacoinvasive, MCS: Mechanical Circulatory Support

Table S4. Management Strategies in Patients with Non-AMI Cardiogenic Shock, Stratified by SCAI and Gender

Non-AMI-CS		SCAI E	SCAI E	P-value	SCAI D	SCAI D	P-value	SCAI C	SCAI C	P-value	SCAI B	SCAI B	P-value
		**Men**	**Women**		**Men**	**Women**		**Men**	**Women**		**Men**	**Women**	
**Mechanical ventilation (%)**		214 (49.4)	182 (49.1)	0.918	110 (35.3)	93 (42.7)	0.084	121 (23.2)	101 (23.6)	0.880	37 (3.2)	41 (4.2)	0.189
**PAC (%)**		31 (7.20)	18 (4.90)	0.173	23 (7.40)	4 (1.80)	0.004	8 (1.50)	2 (0.50)	0.199	2 (0.2)	1 (0.1)	1.00
**Hemodialysis (%)**		72 (16.6)	56 (15.1)	0.553	37 (11.9)	15 (6.90)	0.058	23 (4.40)	22 (5.10)	0.596	25 (2.1)	26 (2.7)	0.41
**MCS (%)**		19 (4.40)	14 (3.80)	0.662	14 (4.50)	7 (3.20)	0.459	17 (3.30)	6 (1.40)	0.064	0 (0.0)	0 (0.0)	NA
**Number of vasoactives (%)**	**0**	84 (19.4)	81 (21.8)	0.007	23 (7.40)	21 (9.60)	0.508	118 (22.6)	91 (21.3)	0.637	1165 (100)	965 (100)	NA
	**1**	68 (15.7)	66 (17.8)		26 (8.30)	14 (6.40)		404 (77.4)	337 (78.7)		0 (0.0)	0 (0.0)	
	**2**	86 (19.9)	100 (27.0)		263 (84.3)	183 (83.9)		0 (0.00)	0 (0.00)		0 (0.0)	0 (0.0)	
	**3**	162 (37.4)	110 (29.6)		0 (0.00)	0 (0.00)		0 (0.00)	0 (0.00)		0 (0.0)	0 (0.0)	
	**4**	33 (7.60)	14 (3.80)		0 (0.00)	0 (0.00)		0 (0.00)	0 (0.00)		0 (0.0)	0 (0.0)	
**Levosimendan (%)**		76 (17.6)	32 (8.60)	<0.001	46 (14.7)	14 (6.40)	0.003	27 (5.20)	5 (1.20)	0.001	0 (0.0)	0 (0.0)	NA
**Dobutamine (%)**		227 (52.4)	145 (39.1)	<0.001	163 (52.2)	76 (34.9)	<0.001	158 (30.3)	91 (21.3)	0.002	0 (0.0)	0 (0.0)	NA
**Norepinephrine (%)**		328 (75.8)	280 (75.5)	0.927	241 (77.2)	187 (85.8)	0.014	213 (40.8)	227 (53.0)	<0.001	0 (0.0)	0 (0.0)	NA
**Vasopressin (%)**		227 (52.4)	195 (52.6)	0.969	102 (32.7)	103 (47.2)	0.001	6 (1.10)	14 (3.30)	0.023	0 (0.0)	0 (0.0)	NA

AMI: Acute Myocardial Infarction, CS: Cardiogenic Shock, SCAI: Society for Cardiovascular Angiography and Interventions, pPCI: Primary Percutaneous Coronary Intervention, PI: Pharmacoinvasive, MCS: Mechanical Circulatory Support

Table S5: Univariate Hazard Ratios for Various Clinical Parameters in Patients with AMI and non-AMI Complicated by CS by Sex

Hazard Ratio	Men AMI-CS		Women AMI-CS		Men Non-AMI-CS		Women Non-AMI-CS	
**Age**	1.03 (1.03-1.04)	<0.001	1.03 (1.02-1.05)	<0.001	1.0 (0.99-1.01)	0.095	1.01 (1.0-1.01)	0.03
**Body mass index**	0.96 (0.95-0.98)	<0.001	0.98 (0.96-1.01)	0.173	0.98 (0.97-1.0)	0.057	0.98 (0.96-0.99)	0.004
**Smoking history**	0.94 (0.8-1.1)	0.943	0.87 (0.66-1.15)	0.866	0.87 (0.74-1.02)	0.078	1.1 (0.88-1.38)	0.389
**Dyslipidemia**	0.95 (0.8-1.13)	0.558	0.78 (0.58-1.03)	0.078	0.88 (0.71-1.09)	0.246	0.92 (0.71-1.2)	0.556
**Hypertension**	1.18 (1.01-1.37)	0.038	0.97 (0.73-1.28)	0.831	0.97 (0.82-1.14)	0.707	0.91 (0.77-1.08)	0.287
**COPD**	1.94 (1.31-2.88)	0.001	1.07 (0.55-2.07)	0.853	1.1 (0.78-1.54)	0.596	1.49 (1.67-1.91)	0.001
**Heart failure history**	1.35 (1.07-1.69)	0.01	1.26 (0.88-1.81)	0.216	1.06 (0.9-1.25)	0.501	1.35 (1.14-1.6)	<0.001
**Chronic Kidney Disease**	2.07 (1.65-2.61)	<0.001	0.93 (0.61-1.41)	0.73	1.23 (1.00-1.5)	0.047	1.22 (0.96-1.55)	0.1
**Diabetes Mellitus**	1.61 (1.38-1.87)	<0.001	1.44 (1.1-1.89)	0.009	0.93 (0.76-1.13)	0.928	0.96 (0.79-1.18)	0.716
**Previous MI**	1.1 (0.92-1.31)	0.308	0.72 (0.51-1.01)	0.059	1.28 (1.05-1.55)	0.014	1.12 (0.81-1.56)	0.494
**Previous PCI**	0.88 (0.69-1.13)	0.312	0.41 (0.21-0.8)	0.009	1.17 (0.89-1.53)	0.256	1.15 (0.71-1.87)	0.564
**Previous CABG**	1.0 (0.62-1.63)	0.987	0.97 (0.48-1.96)	0.93	0.97 (0.65-1.45)	0.887	1.01 (0.52-1.96)	0.973
**Stroke History**	1.31 (0.88-1.94)	0.18	0.99 (0.46-2.11)	0.979	1.42 (1.05-1.94)	0.025	1.29 (0.98-1.7)	0.07
**Previous atrial fibrillation**	1.02 (0.61-1.7)	0.954	1.71 (1.0-2.94)	0.51	1.1 (0.9-1.35)	0.348	1.41 (1.18-1.67)	<0.001
**Type of ACS- STEMI**	1.45 (1.21-1.73)	<0.001	1.48 (1.13-1.94)	0.005	NA	NA	NA	NA
**Systolic blood pressure-▲10 mmHg**	0.79 (0.77-0.81)	<0.001	0.85 (0.82-0.89)	<0.001	0.89 (0.87-0.92)	<0.001	0.89 (0.86-0.92)	<0.001
**Diastolic blood pressure-▲10 mmHg**	0.7 (0.67-0.73)	<0.001	0.72 (0.66-0.78)	<0.001	0.85 (0.81-0.89)	<0.001	0.87 (0.83-0.92)	<0.001
**Medium arterial pressure-▲10 mmHg**	0.71 80.68-0.74)	<0.001	0.76 (0.71-0.81)	<0.001	0.85 (0.81-0.89)	<0.001	0.86 (0.82-0.9)	<0.001
**Heart rate-▲10 lpm**	1.09 (1.06-1.12)	<0.001	1.01 (0.95-1.079	0.78	1.04 (1.01-1.06)	0.002	1.05 (1.02-1.07)	<0.001
**Respiratory rate▲1 bpm**	1.0 (1.0-1.0)	0.284	1.02 (1.01-1.03)	0.005	1.0 (1.0-1.0)	0.123	1.0 (1.0-1.0)	0.88
**LVEF-▲10%**	0.56 (0.52-0.6)	<0.001	0.6 (0.54-0.67)	<0.001	0.92 (0.87-0.96)	<0.001	1.0 (0.99-1.01)	0.866
**Hemoglobin-▲1 g/dL**	0.89 (0.87-0.92)	<0.001	0.97 (0.92-1.03)	0.385	0.96 (0.94-0.99)	0.004	0.99 (0.97-1.02)	0.686
**Leukocytes-▲1 g/dL**	1.06 (1.04-1.07)	<0.001	1.03 (1.01-1.06)	0.007	1.04 (1.03-1.05)	<0.001	1.02 (1.01-1.03)	<0.001
**Neutrophils-▲1 c*10^{9}L**	1.0 (1.0-1.0)	0.611	1.01 (0.98-1.05)	0.426	1.43 (1.32-1.56)	<0.001	1.0 (1.0-1.01)	0.87

**Platelets-▲10 c*10^9L**		0.98 (0.97-0.99)	<0.001	0.97 (0.95-0.98)	<0.001	0.97 (0.96-0.98)	<0.001	0.96 (0.95-0.97)	<0.001
**Glucose-▲25 mg/dL**		1.07 (1.05-1.09)	<0.001	1.04 (1.01-1.06)	0.007	1.01 (0.99-1.04)	0.279	1.01 (0.99-1.04)	0.215
**BUN-▲25 mg/dL**		1.39 (1.33-1.46)	<0.001	1.37 (1.21-1.55)	<0.001	1.22 (1.16-1.29)	<0.001	1.22 (1.16-1.29)	<0.001
**Creatinine -▲1 mg/dL**		1.13 (1.1-1.15)	<0.001	1.14 (1.08-1.21)	<0.001	1.08 (1.05-1.1)	<0.001	1.11 (1.07-1.15)	<0.001
**eGFR-▲ 10 mL/min/1.73m^2**		0.87 (0.76-0.8)	<0.001	0.85 (0.82-0.89)	<0.001	0.89 (0.87-0.92)	<0.001	0.89 (0.87-0.92)	<0.001
**Chloride-▲10 mEq/L**		0.98 (0.94-1.13)	0.754	0.85 (0.68-1.08)	0.187	0.72 (0.64-0.81)	<0.001	0.78 (0.69-0.88)	<0.001
**Sodium-▲10 mEq/L**		0.9 (0.76-1.07)	0.224	0.89 (0.69-1.14)	0.343	0.73 (0.64-0.82)	<0.001	0.86 (0.76-0.97)	0.014
**Potassium -▲1 mEq/L**		1.52 (1.38-1.68)	<0.001	1.34 (1.14-1.58)	<0.001	1.15 (1.06-1.25)	0.001	1.18 (1.09-1.28)	<0.001
**Albumin-▲1 g/dL**		1.01 (1.01-1.02)	<0.001	0.59 (0.45-0.77)	<0.001	0.71 (0.61-0.82)	<0.001	0.6 (0.52-0.7)	<0.001
**AST-▲50 UI/L**		1.01 (1.01-1.02)	<0.001	1.02 (1.01-1.03)	<0.001	1.0 (1.0-1.0)	<0.001	1.01 (1.01-1.01)	<0001
**ALT-▲50 UI/L**		1.02 (1.02-1.02)	<0.001	1.02 (1.01-1.02)	<0.001	1.01 (1.0-1.02)	0.013	1.01 (1.0-1.02)	0.018
**Lactate dehydrogenase-▲100 UI/L**		1.02 (1.01-1.02)	<0.001	1.01 (1.0-1.02)	<0.001	1.01 (1.01-1.02)	<0.001	1.05 (1.04-1.06)	<0.001
**C-Reactive protein-▲ 10 mg/L**		1.04 (1.03-1.05)	<0.001	1.03 (1.02-1.05)	<0.001	1.04 (1.03-1.05)	<0.001	1.04 (1.03-1.05)	<0.001
**72h- Minimum pH▲0.1**		1.0 (1.0-1.0)	0.824	1.01 (1.0-1.01)	<0.001	0.63 (0.56-0.7)	<0.001	0.67 (0.59-0.76)	<0.001
**72h- Maximum lactate▲1 mmol/L**		1.26 (1.23-1.27)	<0.001	1.23 (1.19-1.26)	<0.001	1.15 (1.13-1.17)	<0.001	1.04 (1.03-1.04)	<0.001
**Primary reperfusion (%)**	**NR***	*Reference*		*Reference*		NA	NA	NA	NA
	**pPCI**	0.69 (0.56-0.84)	<0.001	0.85 (0.6-1.21)	0.366				
	**PI**	0.62 (0.44-0.86)	0.004	0.74 (0.4-1.35)	0.321				
**PAC (%)**		2.59 (2.12-3.11)	<0.001	1.52 (1.03-2.24)	0.036	1.9 (1.31-2.75)	0.001	1.39 (0.77-2.53)	0.277
**Mechanical ventilation (%)**		7.6 (6.47-8.93)	<0.001	4.49 (3.49-5.79)	<0.001	4.11 (3.49-4.84)	<0.001	3.26 (2.75-3.86)	<0.001
**Hemodialysis (%)**		301 (2.39-3.78)	<0.001	1.69 (1.13-2.52)	0.01	1.89 (1.49-2.4)	<0.001	1.38 (1.04-1.83)	0.027
**Number of vasoactives (%)**	**0**	*Reference*		*Reference*		*Reference*		*Reference*	
	**1**	4.53 (3.44-5.97)	<0.001	4.0 (2.64-6.05)	<0.001	2.77 (2.17-3.52)	<0.001	2.32 (1.82-2.96)	<0.001
	**2**	9.52 (7.43-12.19)	<0.001	7.91 (5.34-11.72)	<0.001	5.63 (4.48-7.07)	<0.001	5.89 (4.7-7.38)	<0.001
	**3**	16.24 (12.85-20.52)	<0.001	12.35 (8.4-18.14)	<0.001	8.81 (6.85-11.34)	<0.001	8.07 (6.16-10.57)	<0.001
	**4**	14.44 (10.64-19.59)	<0.001	10.26 (6.06-17.36)	<0.001	7.43 (4.68-11.8)	<0.001	4.38 (2.22-8.63)	<0.001
**Levosimendan (%)**		2.53 (2.06-3.11)	<0.001	2.5 (1.7-3.69)	<0.001	1.2 (0.9-1.6)	0.216	1.45 (0.95-2.22)	0.089

**Dobutamine (%)**		5.08 (4.34-5.96)	<0.001	3.87 (3.0-4.99)	<0.001	1.89 (1.6-2.32)	<0.001	1.97 (1.63-2.39)	<0.001
**Norepinephrine (%)**		7.84 (6.54-9.4)	<0.001	6.5 (4.83-8.76)	<0.001	5.45 (4.56-6.52)	<0.001	4.53 (3.76-5.45)	<0.001
**Vasopressin (%)**		7.97 (6.82-9.31)	<0.001	5.62 (4.36-7.23)	<0.001	5.84 (4.96-6.89)	<0.001	4.87 (4.1-5.78)	<0.001
**MCS (%)**		3.72 (3.18-4.35)	<0.001	1.99 (1.48-2.68)	<0.001	1.1 (0.64-1.86)	0.738	0.91 (0.45-1.82)	0.906
**SCAI (%)**	**E**	24.15 (18.31-31.85)	<0.001	15.24 (10.16-22.86)	<0.001	8.39 (6.59-10.67)	<0.001	7.83 (6.16-9.95)	<0.001
	**D**	12.25 (9.05-16.59)	<0.001	6.89 (4.34-10.93)	<0.001	4.86 (3.72-6.36)	<0.001	4.93 (3.7-6.51)	<0.001
	**C**	5.2 (3.74-7.25)	<0.001	3.72 (2.32-5.99)	<0.001	2.57 (1.96-3.38)	<0.001	2.06 (1.55-2.73)	<0.001
	**B**	*Reference*		*Reference*		*Reference*		*Reference*	

*NR patients comprise NSTEMI patients or STEMI late presenters who did not have a primary reperfusion

ACS: Acute Coronary Syndrome, AMI: Acute Myocardial Infarction, ALT: Alanine Aminotransferase, AST: Aspartate Aminotransferase, BUN: Blood Urea Nitrogen, CABG: Coronary Artery Bypass Grafting, COPD: Chronic Obstructive Pulmonary Disease, eGFR: Estimated Glomerular Filtration Rate, LVEF: Left Ventricular Ejection Fraction, NR: Not primary reperfused, PCI: Percutaneous Coronary Intervention, pPCI: Primary PCI, PI: pharmacoinvasive strategy, SCAI: Society for Cardiovascular Angiography and Interventions, STEMI: ST-Elevation Myocardial Infarction

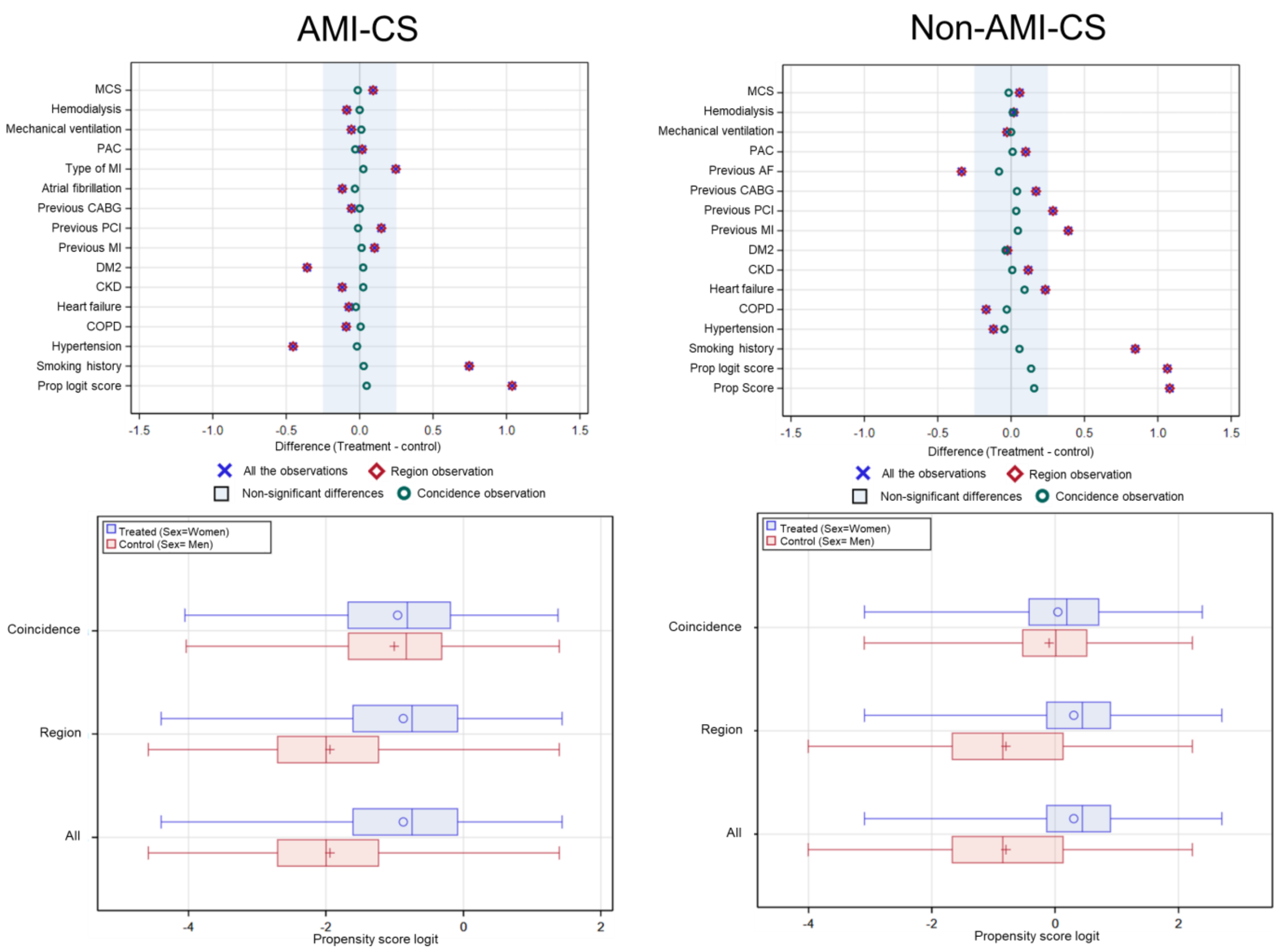

Figure S3. Propensity score matching baseline characteristics (AMI-CS and non-AMI-CS), and distribution of propensity scores logit (bottom). CABG: Coronary Artery Bypass Grafting, CKD: Chronic Kidney Disease, COPD: Chronic Obstructive Pulmonary Disease, DM2: Diabetes Mellitus Type 2, MI: Myocardial Infarction, MCS: Mechanical Circulatory Support, PAC: Pulmonary Artery Catheterization, PCI: Percutaneous Coronary Intervention

Table S6. Demographics, Clinical Parameters, Management, and Outcomes of Patients with AMI and non-AMI Cardiogenic Shock, Stratified by Sex in the Propensity Score Matched Cohort.

		AMI-CS			non-AMI-CS		
		Men (n=931)	Women (n=931)	P-value	Men (n=1406)	Women (n=1406)	P-value
**Age (years)**		65 (57-73)	66 (58-74)	0.07	60 (45-73)	61 (46-73)	0.246
**Body mass index (Kg/m^2)**		26.77 (24.45-29.38)	26.64 (23.88-29.73)	0.382	25.61 (23.31-28.37)	25.39 (22.31-29.28)	0.31
**Smoking history (%)**		330 (35.4)	318 (34.2)	0.559	348 (24.8)	313 (22.3)	0.12
**Dyslipidemia (%)**		271 (29.1)	280 (30.1)	0.648	214 (15.2)	199 (14.2)	0.424
**Hypertension (%)**		636 (68.3)	644 (69.2)	0.689	604 (43)	636 (45.2)	0.224
**COPD (%)**		28 (3.00)	27 (2.90)	0.891	95 (6.8)	106 (7.5)	0.421
**Heart failure history (%)**		93 (10.0)	100 (10.7)	0.595	686 (48.8)	622 (44.2)	0.016
**Chronic Kidney Disease (%)**		92 (9.90)	86 (9.20)	0.636	194 (13.8)	190 (13.5)	0.826
**Diabetes Mellitus (%)**		544 (58.4)	533 (57.3)	0.606	236 (23.2)	349 (24.8)	0.31
**Previous MI (%)**		185 (19.9)	180 (19.3)	0.77	156 (11.1)	134 (9.5)	0.173
**Previous PCI (%)**		75 (8.10)	78 (8.40)	0.8	59 (4.9)	57 (4.1)	0.274
**Previous CABG (%)**		31 (3.30)	31 (3.30)	1.0	39 (2.8)	29 (2.1)	0.22
**Stroke History (%)**		43 (4.60)	24 (2.60)	0.018	101 (7.2)	106 (7.5)	0.718
**Previous atrial fibrillation (%)**		30 (3.20)	35 (3.80)	0.528	325 (23.1)	375 (26.7)	0.029
**Type of ACS (%)**	**NSTEMI**	333 (35.8)	344 (36.9)	0.596	(-)	(-)	NA
	**STEMI**	598 (64.2)	587 (63.1)		(-)	(-)	
**Admission vital signs and paraclinical work-up**							
**Systolic blood pressure (mmHg)**		122 (104-142)	122 (100-145)	0.706	110 (90-130)	110 (90-130)	0.435
**Diastolic blood pressure (mmHg)**		75 (63-90)	73 (60-86)	0.014	66 (60-80)	64 (59-80)	0.301
**Medium arterial pressure (mmHg)**		90 (79.33-105.33)	90 (76.67-104.33)	0.143	80.67 (70-93.33)	80 (70-93.33)	0.432
**Heart rate (bpm)**		80 (68-98)	80 (68-98)	0.724	90 (70-110)	90 (68-110)	0.531
**Respiratory rate (rpm)**		18 (17-20)	18 (17-22)	0.128	20 (18-24)	20 (18-24)	0.73
**LVEF (%)**		45 (35-54)	45 (35-55)	<0.001	45 (27-59)	52.5 (35-60)	<0.001
**Hemoglobin (g/dL)**		14.8 (13-16.1)	13.1 (11.6-14.4)	<0.001	14 (12-15.7)	12.6 (10.3-14.3)	<0.001

**Leukocytes (c*10^{9}L)**		11.16 (8.7-14.3)	11 (8.7-13.89)	0.362	9.7 (7.4-12.9)	9.6 (7-13.5)	0.264
**Neutrophils (%)**		78.65 (70-85)	77.85 (68.3-84)	0.043	78 (69.2-85)	78.55 (69-86)	0.627
**Platelets (c*109L)**		216 (179-268)	254.5 (205-303)	<0.001	184 (140-243)	195 (142-265)	<0.001
**Glucose (mg/dL)**		174 (130-250)	179 (130-268.8)	0.218	117 (98-152)	124 (100-166)	0.001
**BUN (mg/dL)**		22 (16-34)	22 (15.7-33.05)	0.349	31 (20-53)	29 (17-49)	0.001
**Creatinine (mg/dL)**		1.2 (0.93-1.7)	1 (0.76-1.5)	<0.001	1.4 (1.03-2.2)	1.2 (0.87-1.91)	<0.001
**eGFR (mL/min/1/. 73m^2)**		64.42 (40.56-85.34)	58.68 (35.51-83.77)	0.043	53.26 (30.32-80.58)	50.28 (26.38-77.61)	0.013
**Chloride (mEq/L)**		103 (100-106)	102 (98.75-105)	<0.001	101 (97-105)	101 (96-105)	0.814
**Sodium (mEq/L)**		136 (134-139)	136 (133-139)	0.141	135.8 (131.4-139)	135 (131-138.2)	0.169
**Potassium (mEq/L)**		4.3 (3.9-4.7)	4.27 (3.9-4.7)	0.341	4.5 (4-5.1)	4.4 (3.93-5.1)	0.017
**Albumin (g/dL)**		3.63 (3.27-3.99)	3.55 (3.2-3.88)	0.001	3.4 (2.98-3.8)	3.4 (3-3.8)	0.859
**AST (UI/L)**		71 (32-218.3)	64 (30-188)	0.083	36 (23-81)	35 (22.8-60.1)	0.043
**ALT (UI/L)**		47.95 (29-89)	41 (23-84)	<0.001	30.79 (19-69.15)	27.2 (17-54.3)	<0.001
**Lactate dehydrogenase (UI/L)**		445 (244-860)	430.5 (250-794.5)	0.685	350 (217-589)	337 (218-582)	0.794
**C-Reactive protein (mg/L)**		14 (4.63-72.2)	14.2 (4.6-64)	0.711	28 (8.7-87.85)	22.15 (7.4-68.41)	0.006
**72h- Minimum pH**		7.39 (7.33-7.43)	7.39 (7.33-7.43)	0.785	7.38 (7.32-7.44)	7.38 (7.31-7.44)	0.18
**72h- Maximum lactate**		2.4 (2-3.4)	2.3 (2-3.4)	0.434	2.4 (2-3.6)	2.4 (1.8-3.6)	0.126
**Cardiogenic shock management**							
**Primary reperfusion (%)**	**NR***	655 (70.4)	663 (71.2)	0.632	(-)	(-)	NA
	**pPCI**	199 (21.4)	202 (21.7)		(-)	(-)	
	**PI**	77 (8.3)	66 (7.1)		(-)	(-)	
**PAC (%)**		56 (6)	63 (6.8)	0.507	24 (1.7)	22 (1.6)	0.766
**Mechanical ventilation (%)**		173 (18.6)	169 (18.2)	0.811	291 (20.7)	291 (20.7)	1.0
**Hemodialysis (%)**		49 (5.3)	49 (5.30)	1.0	86 (6.1)	82 (5.8)	0.75
**Number of vasoactives**	**0**	574 (61.7)	576 (61.9)	0.372	820 (58.3)	806 (57.3)	0.68
	**1**	156 (16.8)	143 (15.4)		281 (20)	298 (21.2)	
	**2**	94 (10.1)	103 (11.1)		196 (13)	207 (14.7)	
	**3**	91 (9.8)	82 (8.80)		93 (6)	84 (6.0)	
	**4**	16 (1.7)	27 (2.90)		16 (1)	11 (0.8)	

**Levosimendan (%)**		50 (5.4)	41 (4.40)	0.33	85 (6.0)	45 (3.2)	<0.001
**Dobutamine (%)**		220 (23.6)	212 (22.8)	0.661	288 (20.5)	243 (17.3)	0.03
**Norepinephrine (%)**		289 (31.0)	309 (33.2)	0.321	451 (32.1)	493 (35.1)	0.094
**Vasopressin (%)**		122 (13.1)	141 (15.1)	0.206	192 (13.7)	227 (16.1)	0.064
**MCS (%)**		111 (11.9)	115 (12.4)	0.777	22 (1.6)	25 (1.8)	0.659
**Cardiogenic Shock severity**							
**SCAI (%)**	**E**	151 (16.2)	151 (16.2)	1.0	248 (17.6)	248 (17.6)	1.0
	**D**	105 (11.3)	105 (11.3)		175 (12)	175 (12.4)	
	**C**	164 (17.6)	164 (17.6)		299 (21)	299 (21.3)	
	**B**	511 (54.9)	511 (54.9)		684 (48)	684 (48.6)	
**Cardiogenic Shock Outcomes**							
**Mortality (%)**		179 (19.2)	220 (23.6)	0.021	348 (24.8)	367 (26.1)	0.411
**Stroke (%)**		17 (1.80)	20 (2.1)	0.618	19 (1.4)	22 (1.6)	0.637
**VT / VF (%)**		129 (13.9)	116 (12.5)	0.373	128 (9.1)	132 (9.4)	0.795
**GIB (%)**		27 (2.90)	25 (2.7)	0.778	32 (2.3)	35 (2.5)	0.711
**PE (%)**		0	1 (0.1)	1.0	5 (0.4)	7 (0.5)	0.593
**AKI (%)**		253 (27.2)	275 (29.5)	0.258	528 (37.6)	524 (37.3)	0.876
**Nosocomial pneumonia (%)**		47 (5.00)	39 (4.2)	0.377	47 (3.3)	55 (3.9)	0.42
**Sepsis (%)**		35 (3.80)	38 (4.1)	0.72	123 (8.7)	101 (7.2)	0.125

*NR patients comprise NSTEMI patients or STEMI late presenters who did not have a primary reperfusion

ACS: Acute Coronary Syndrome, AMI: Acute Myocardial Infarction, AKI: Acute Kidney Injury, ALT: Alanine Aminotransferase, AST: Aspartate Aminotransferase, BUN: Blood Urea Nitrogen, CABG: Coronary Artery Bypass Grafting, COPD: Chronic Obstructive Pulmonary Disease, CRP: C-Reactive Protein, CS: Cardiogenic Shock, eGFR: Estimated Glomerular Filtration Rate, GIB: Gastrointestinal Bleeding, LVEF: Left Ventricular Ejection Fraction, MCS: Mechanical Circulatory Support, NSTEMI: Non-ST Segment Elevation Myocardial Infarction, NR: Not primary reperfused, PCI: Percutaneous Coronary Intervention, primary PCI (pPCI), PE: Pulmonary Embolism, PAC: Pulmonary Artery Catheterization, PI: pharmacoinvasive strategy, SCAI: Society for Cardiovascular Angiography and Interventions, STEMI: ST Segment Elevation Myocardial Infarction, VF: Ventricular Fibrillation, VT: Ventricular Tachycardia.
